# Mammalian Hbs1L deficiency causes congenital anomalies and developmental delay associated with Pelota depletion and 80S monosome accumulation

**DOI:** 10.1371/journal.pgen.1007917

**Published:** 2019-02-01

**Authors:** Amy E. O’Connell, Maxim V. Gerashchenko, Marie-Francoise O’Donohue, Samantha M. Rosen, Eric Huntzinger, Diane Gleeson, Antonella Galli, Edward Ryder, Siqi Cao, Quinn Murphy, Shideh Kazerounian, Sarah U. Morton, Klaus Schmitz-Abe, Vadim N. Gladyshev, Pierre-Emmanuel Gleizes, Bertrand Séraphin, Pankaj B. Agrawal

**Affiliations:** 1 Division of Newborn Medicine, Boston Children’s Hospital, Boston, Massachusetts, United States of America; 2 Department of Pediatrics, Harvard Medical School, Boston, Massachusetts, United States of America; 3 Division of Genetics, Department of Medicine, Brigham and Women’s Hospital, Harvard Medical School, Boston, Massachusetts, United States of America; 4 Laboratoire de Biologie Moléculaire Eucaryote, Centre de Biologie Intégrative, Université de Toulouse, CNRS, UPS, Toulouse, France; 5 Division of Genetics and Genomics, Boston Children’s Hospital, Boston, Massachusetts, United States of America; 6 The Manton Center for Orphan Disease Research, Boston Children’s Hospital, Boston, Massachusetts, United States of America; 7 Institut de Génétique et de Biologie Moléculaire et Cellulaire, Illkirch, France; 8 Université de Strasbourg, Centre National de La Recherche Scientifique UMR 7104, INSERM U964, Strasbourg, France; 9 Wellcome Sanger Institute, Cambridge, United Kingdom; 10 Broad Institute of MIT and Harvard, Cambridge, Massachusetts, United States of America; Stanford University School of Medicine, UNITED STATES

## Abstract

Hbs1 has been established as a central component of the cell’s translational quality control pathways in both yeast and prokaryotic models; however, the functional characteristics of its human ortholog (Hbs1L) have not been well-defined. We recently reported a novel human phenotype resulting from a mutation in the critical coding region of the *HBS1L* gene characterized by facial dysmorphism, severe growth restriction, axial hypotonia, global developmental delay and retinal pigmentary deposits. Here we further characterize downstream effects of the human *HBS1L* mutation. *HBS1L* has three transcripts in humans, and RT-PCR demonstrated reduced mRNA levels corresponding with transcripts V1 and V2 whereas V3 expression was unchanged. Western blot analyses revealed Hbs1L protein was absent in the patient cells. Additionally, polysome profiling revealed an abnormal aggregation of 80S monosomes in patient cells under baseline conditions. RNA and ribosomal sequencing demonstrated an increased translation efficiency of ribosomal RNA in Hbs1L-deficient fibroblasts, suggesting that there may be a compensatory increase in ribosome translation to accommodate the increased 80S monosome levels. This enhanced translation was accompanied by upregulation of mTOR and 4-EBP protein expression, suggesting an mTOR-dependent phenomenon. Furthermore, lack of Hbs1L caused depletion of Pelota protein in both patient cells and mouse tissues, while *PELO* mRNA levels were unaffected. Inhibition of proteasomal function partially restored Pelota expression in human Hbs1L-deficient cells. We also describe a mouse model harboring a knockdown mutation in the murine *Hbs1l* gene that shared several of the phenotypic elements observed in the Hbs1L-deficient human including facial dysmorphism, growth restriction and retinal deposits. The *Hbs1l*KO mice similarly demonstrate diminished Pelota levels that were rescued by proteasome inhibition.

## Author summary

We have identified a patient with biallelic mutations in *HBS1L* affecting Hbs1LV1 and V2 transcripts leading to a loss of Hbs1L implicated in ribosomal recycling. In contrast to yeast studies, loss of Hbs1LV1/V2 in human cells did not appear to impact the translational quality control mechanisms of non-stop and no-go decay. However, patient cells demonstrated accumulation of free 80S ribosomes based on polysome profiling. In addition, Hbs1LV1/V2 deficient cells demonstrated an increase in translation efficiency of ribosomal mRNA based on RiboSeq data, which may suggest an attempt to compensate for defective mobilization of free 80S ribosomes. The patient samples demonstrated increased 4EBP1 and mTOR expression and phosphorylation compared to controls, suggesting an mTOR-dependent ribosomal RNA regulation is involved in the response to Hbs1LV1/V2 deficiency. Loss of Hbs1L in both human and mouse fibroblasts lead to diminished Pelota levels, and this phenomenon could be partially rescued by proteasome inhibition. In all, these data support a role for Hbs1LV1/V2 as a Pelota binding partner with a specific function in utilization of free ribosomes.

## Introduction

Hbs1L belongs to a specialized family of translational GTPases (trGTPases), members of which are structurally homologous but functionally distinct [1]. Each trGTPase binds to a specific “decoding” protein and transports it to the ribosomal A site, where it recognizes a unique mRNA code. In mammals, eEF1A transports aminoacyl (aa)-tRNAs to sense codons, eRF3 transports eRF1 to termination codons, and Hbs1L transports Pelota to stalled ribosomes with either an empty A site or an mRNA-occupied A site without sequence preference [2, 3]. Engagement of each decoding protein with the ribosome initiates a distinct anabolic event: aa-tRNAs lengthen the nascent chain, eRF1 terminates translation, and Pelota triggers mRNA surveillance pathways.

mRNA surveillance is a critical component of translational quality control (tQC) in all cells. There are three mRNA surveillance pathways that have been well-defined in eukaryotes, each of which is responsible for the selective degradation of a specific class of aberrant mRNA. Nonsense-mediated decay (NMD) targets sequences containing a premature termination codon [4], non-stop decay (NSD) degrades mRNAs lacking any termination codon [5, 6], and no-go decay (NGD) targets mRNAs containing cis-acting features that cause translational arrest [7]. Pelota:Hbs1L has been implicated in NGD and NSD in plants and eukaryotes [7–11]. Our understanding of its role in these processes is largely predicated on studies in *Saccharomyces cerevisiae* of the orthologous protein complex, Dom34:Hbs1. Yeast Hbs1 (Hsp70 subfamily B suppressor 1) was originally identified for its ability to rescue stalled ribosomes by suppressing Hsp70 (heat shock protein 70) activity [12]. Subsequent studies structurally associated Hbs1 with eRF3 and eEF1A [13], and recognition of Dom34 as an Hbs1-interacting protein functionally linked the complex to translation [14]. Recent biochemical studies have shown that Dom34:Hbs1 promotes the dissociation of the stalled ribosome into subunits [8, 15, 16] during aberrant translation. Subunit dissociation is a critical component of canonical ribosomal recycling, a process to reutilize ribosomes from a terminated or aberrant round of translation.

Hbs1 and Dom34 have additionally been implicated in ribosomal recycling mechanisms outside of canonical tQC pathways. For example, the Dom34:Hbs1 complex has been suggested to play a role in ribosomal recycling of inactive 80S monosomes in yeast cells recovering from stress [17]. Under stress conditions, free ribosomal subunits associate to form large pools of inactive 80S monosomes stabilized by the ‘clamping’ Stm1 factor [17–22]. This effectively limits or inhibits translation, allowing the cell to reserve metabolic resources and protect ribosomal subunits from damage and degradation. Dom34:Hbs1 has been shown to work in conjunction with Rli1 (ABCE1 in mammals) to dissociate inactive ribosomes following stress relief, thus playing a critical role in translation re-initiation during stress recovery [17]. Interestingly, 80S monosome accumulation has been observed in Hbs1- and Dom34- deficient yeast cells even in non-stressed conditions, suggesting that under normal conditions there may be inactive ribosomes that require Dom34:Hbs1-mediated dissociation prior to starting translation [17].

We have previously described an Hbs1L-deficient patient with a phenotype that included severe intrauterine growth restriction, microcephaly, axial hypotonia, lax joints, global developmental delay, fused C2-C3 vertebrae, scoliosis, submucous cleft palate and retinal pigmentary deposits [23]. We have now interrogated Hbs1L-deficient mice that demonstrated a similar phenotype. Our data demonstrate that one of the important functions of Hbs1L in mammals is to stabilize Pelota protein, and proteasomal inhibition can increase Pelota levels in the Hbs1L-deficient cells. Furthermore, Hbs1L:Pelota appear to be important for utilization of free 80S ribosomes, and in the absence of Hbs1L, there is an increase in ribosomal RNA translation efficiency (TE) which may act to compensate for the impaired ability to utilize 80S monosomes.

## Results

### Hbs1L deficiency leads to a unique phenotype featuring growth restriction, facial dysmorphism, and developmental delay

**Fig 1:**
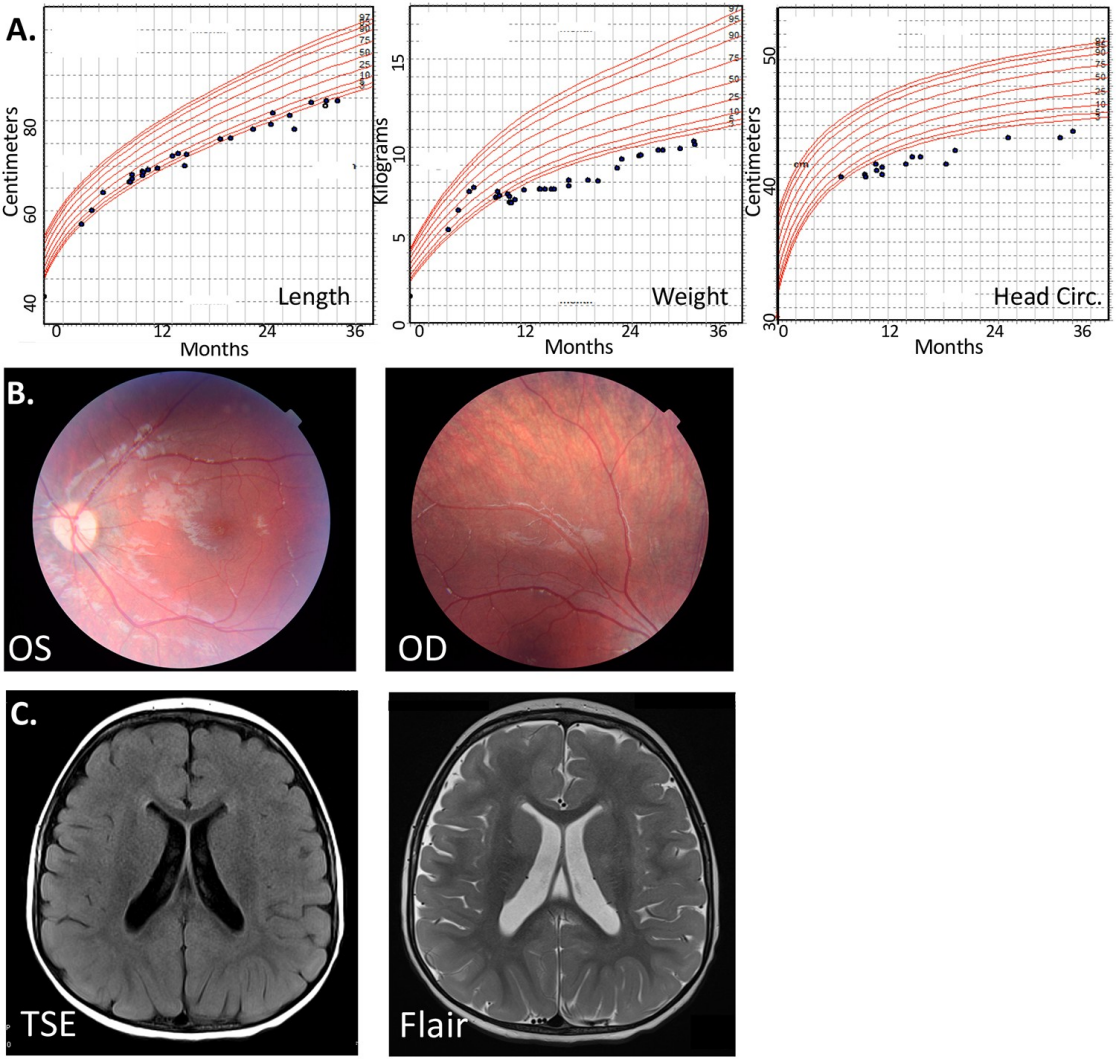
Clinical findings in patient with Hbs1L-V1 and V2 deficiency. (A) Growth restriction was sustained from birth through childhood. References shown are WHO standards. (B) Retinal imaging showing bilateral hyperpigmented deposits. (C) MRI showing prominence of the ventricles and sulci without parenchymal signal abnormalities.

The HBS1L:MYB locus has been linked to a number of hematological traits including fetal hemoglobin levels, red and white blood cell counts, and platelet counts by genome-wide association studies (GWAS) [24–27], and we have previously described a female child with no apparent hematological abnormalities carrying compound heterozygous mutations resulting in Hbs1L deficiency [23]. The child had multiple congenital anomalies (MCA) of unknown molecular basis. These included sparse hair and eyebrows, deep-set eyes with blue sclerae, bifid uvula with a submucous cleft palate, velopharyngeal insufficiency, C2-C3 vertebral fusion, scoliosis, vesicoureteral reflux with a bladder diverticulum, significant hypotonia, and global developmental delay [23]. To delineate the phenotype further, the patient was born at 37 weeks gestation with severe symmetric intrauterine growth restriction, weighing only 1530 grams at birth (<1^st^ percentile, z-score -3.72), head circumference of 29.5 cm (<1^st^ percentile, z-score -2.59) and length of 41 cm (<1^st^ percentile, z-score -2.84). Growth restriction persisted throughout childhood, and at four years old she remained at the 3^rd^ percentile for height, the 1^st^ percentile for weight, and the 0.5^th^ percentile for head circumference (Fig 1A).

In addition to the facial findings described previously, the patient had low posterior hairline, low-set ears, midface hypoplasia, a thin nose with a small mouth and small teeth, and small medial clefts on the lower third of each eyelid. Further issues include dysphagia with aspiration, chronic sinus disease and left-sided conductive hearing impairment needing tympanostomy tube. The patient also displayed thirteen pairs of ribs. She had no history of anemia, abnormal bleeding, or increased infectious susceptibility, and routine complete blood counts revealed normal hematocrit indices and red blood cell, white blood cell, and platelet counts over time. The patient’s mother, father, and younger brother had no medical problems and extensive family history revealed no other family members with similar constellations of symptoms.

The patient’s muscle bulk was greatly diminished throughout the body; axial muscles more affected than appendicular. She had delayed motor milestones as she began to sit unsupported at twelve months and achieved independent ambulation at six years. Her psychological testing at four years demonstrated significant delays in language development and fine and gross motor development, although nonverbal reasoning skills were average. The patient had some astigmatism and decreased visual acuity (20/100 OS and 20/80 OD), and retinal examination revealed diffuse pigmented deposits (Fig 1B).

Magnetic resonance imaging (MRI) at 30 months showed prominence of the ventricles and sulci with rare, scattered non-specific foci of T2 prolongation in the cerebral white matter (Fig 1C). Her electromyography was normal. Renal ultrasound revealed asymmetry (right larger than left) with a duplex right kidney.

### Compound heterozygous HBS1L mutations cause deficiency of Hbs1L-V1 and V2 but not V3

**Fig 2:**
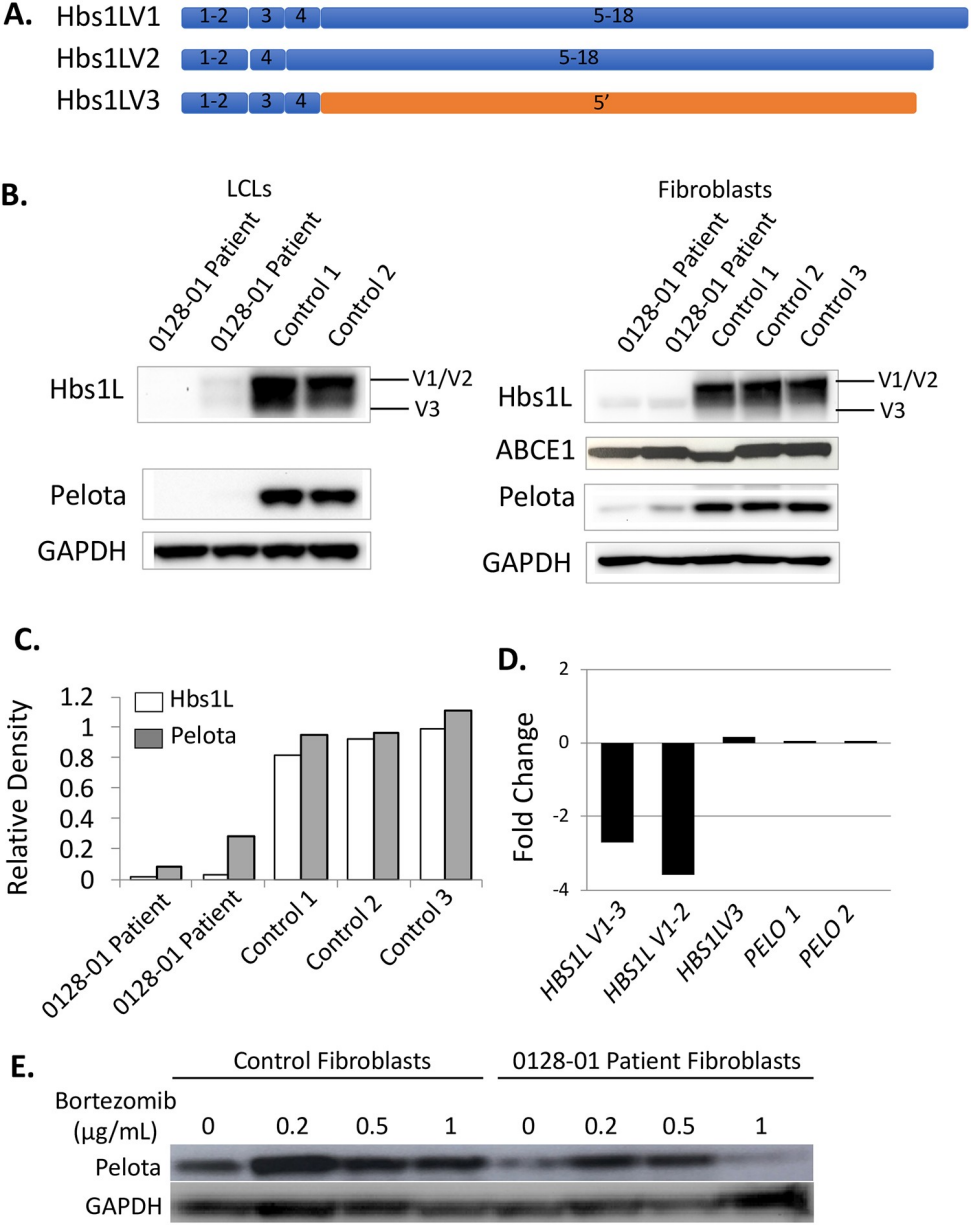
Biallelic mutations in human *HBS1L* lead to Hbs1L deficiency and decreased Pelota protein. (A) Schematic of Hbs1L isoforms V1, V2, V3 indicating exon changes. (B) Western blots probing for Hbs1L, Pelota, ABCE1, and GAPDH protein expression in EBV-transformed lymphocytic cell lines (LCL) and dermal fibroblasts derived from patient and controls. Bands corresponding to Hbs1L isoforms 1/2 and 3 are indicated. (C) Density read-out from Western blot of fibroblasts depicted in B. (D) Relative expression (in fold change versus control) of *HBS1L* transcripts as determined by RT-PCR using primers to HBS1L-V1, V2, and V3 (exons 2–4), primers to HBS1L-V1 and V2 only (exons 15–17), and primers specific to exon 5 of HBS1L-V3, as well as primers to PELO exons 2–3 (PELO 1) and exon 2 (PELO 2). (E) Dose response curve to proteasome inhibition with Bortezomib showing Pelota expression by Western blot for increasing concentrations of Bortezomib in control and Hbs1L-deficient (0128–01) dermal fibroblasts. Each experiment represents a minimum of two technical replicates with highly similar results.

The patient had a normal 46, XX karyotype and a normal chromosomal microarray. Whole-exome sequencing was subsequently performed on the trio as reported previously revealing a null splice donor mutation (hg19; chr6:135287466, c.2043+1G>T) inherited from the father and a nonsense mutation (hg19; chr6:135290431, NM_006620: c.1843C>T:p.R615X) inherited from the mother in *HBS1L* [3, 23]. Western blot analysis revealed no detectable expression of Hbs1L-V1 and V2 proteins in patient fibroblasts and LCLs (Fig 2A–2C), although a band corresponding to the expected location of Hbs1L-V3 [2] was present and was similar to controls. RT-PCR similarly demonstrated decreased *HBS1L* transcript levels when probing for exons 2–4 (V1, V2 and V3), marked reduction in exons 15–17 (only present in V1 and V2), and no change in transcript levels for exon 5 of the V3. This reflects depletion of the *HBS1L-*V1 and V2 variants, and essentially no change in *HBS1L-*V3 transcript levels compared to controls (Fig 2D).

### Pelota expression decreases with Hbs1L deficiency and increases with proteasome inhibition

As Pelota is a known interacting partner of Hbs1L, we evaluated its abundance using Western blot. Pelota levels were markedly reduced in patient fibroblasts relative to controls (Fig 2B–2C). In order to determine whether this change occurred on the messenger RNA (mRNA) level, a reverse transcription polymerase chain reaction (RT-PCR) was used to compare the abundance of *PELO* transcripts in patient and control fibroblasts. *PELO* levels were comparable between them (Fig 2D), indicating that the absence of Hbs1L did not alter *PELO* transcript levels. Western blotting experiments were then repeated in the presence of a 20S proteasome inhibitor to evaluate Pelota stability in absence of Hbs1L. Proteasome inhibition was found to increase abundance of Pelota, drastically increasing at the initial 0.2 μg/mL dose, but then progressively decreased with higher doses of 0.5 and 1.0 μg/mL suggestive of toxic effects at higher doses. This pattern was noted in both patient and control cells (Fig 2E).

### Hbs1L-deficient patient cells have higher baseline levels of 80S ribosomes

**Fig 3:**
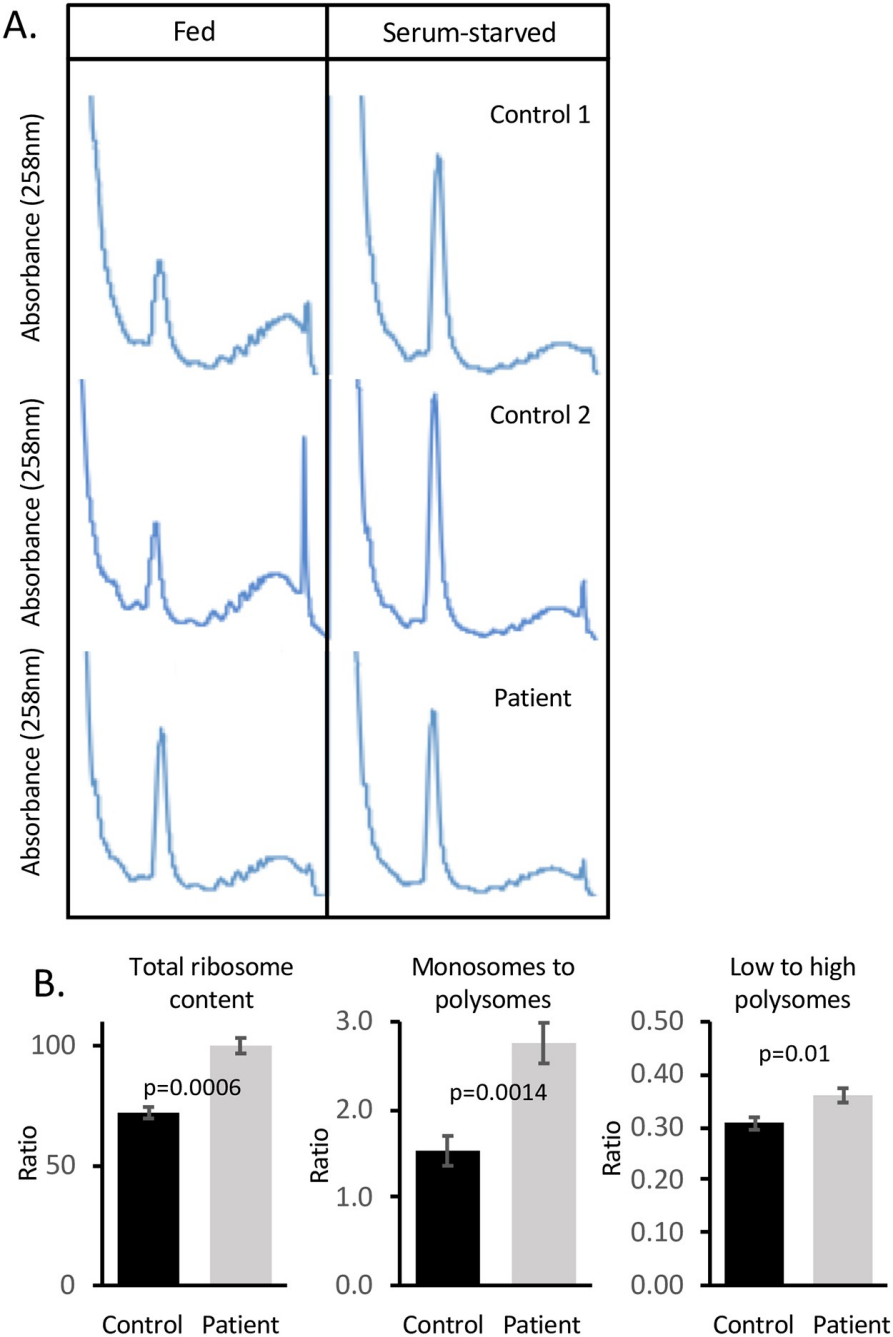
Hbs1L-deficient fibroblasts have elevated 80S ribosomal monosome levels. (A) Polysome profiles of Hbs1L-deficient (bottom panels) and control fibroblasts under fed (+15% FBS) and starved (20–24 hours without FBS) conditions. The polysome profile is representative of 3 independent technical replicates with highly similar results. (B) Ratios of monosomes and polysomes for control and patient fibroblasts. p-value determined by paired t-test.

Hbs1L has been implicated in the recycling of inactive ribosomes after periods of cell stress to promote translational re-initiation [17]. In order to investigate the effect of Hbs1L deficiency on this process, a glucose starvation assay was performed and polysome profiles were assessed on and fibroblasts from patient and control cell lines. Interestingly, patient fibroblasts displayed an increased 80S monosome peak relative to controls under baseline conditions. When the fibroblast samples subsequently underwent starvation, the 80S peak rose in control cells to match the patient baseline level, whereas the patient cells showed no significant changes (Fig 3A). The total ribosome content and the ratio of monosomes to polysomes were increased in the patient fibroblasts (Fig 3B).

### Hbs1L-deficient cells demonstrate normal ribosomal RNAs

**Fig 4:**
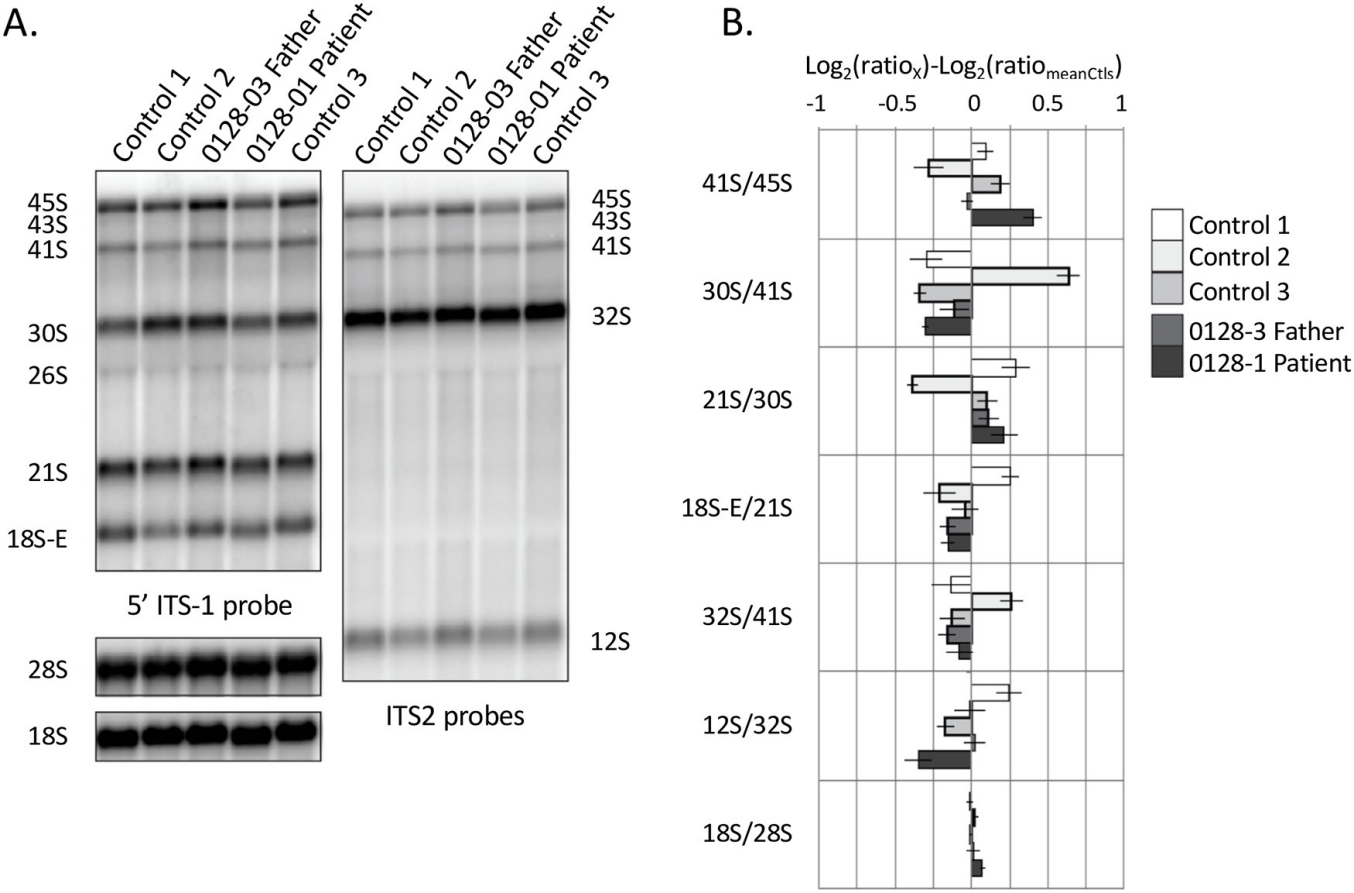
Ribosomal RNA profiles are similar in Hbs1L-deficient and control LCLs. (A) Northern blot analysis of total RNAs extracted from control or Hbs1L-deficient LCLs. Pre-rRNA species were evidenced with 5’ITS1 and ITS2 probes, revealing precursors to the 18S rRNA (component of the small ribosomal subunit) and the 5.8S and 28S rRNAs (components of the large ribosomal subunit), respectively. (B) Pre-rRNAs were quantified and log_2_ ratios of product to precursor pre-rRNA species, or 18S to 28S rRNAs, were calculated and normalized to the mean value obtained for 3 healthy controls (controls 1–3). The data were obtained from three independent experiments (± S.E.M.).

Ribosomal RNAs (rRNAs) are critical for the maturation and assembly of ribosomal subunits. In order to investigate whether Hbs1L exerts influence over ribosomal processing, we analyzed the levels of several 18S rRNA intermediates (45S, 43S, 41S, 30S, 21S, 18S-E) and 28S rRNA intermediates (45S, 43S, 41S, 32S, 12S) in the patient-derived Hbs1L-deficient LCLs and three unrelated control cell lines by Northern blot analysis using probes complementary to the ITS1 and ITS2 sequences, respectively (Fig 4A). Bands for each of these intermediate species were quantified and calculated as product to precursor ratios. In patient cells, 18S precursor pre-rRNA ratios 41S/45S, 30S/41S, 21S/30S, and 18S-E/21S, as well as 28S precursor pre-rRNA ratios 32S/41S and 12S/32S, were similar to controls (Fig 4B). 18S/28S rRNA ratios were also in the same range for patient and control cells, as demonstrated by Western blot analysis (Fig 4A, lower panel).

In addition to patient’s cells, small interfering RNA (siRNA) experiments were used to investigate the role of Hbs1L in rRNA processing. HeLa cells were treated with one of two different anti-*HBS1L* siRNAs: *HBS1L-*1 targeted *HBS1L-*V1, V2, and V3 transcripts, whereas the action of *HBS1L*-2 was restricted to *HBS1L*-V1 and V2, as attested by Western blot (S1 Fig). HeLa cells treated with siRNA *HBS1L*-1 showed some reduction of the 30S/41S ratio compared to controls and a concomitant increase of 21S/30S and 18S-E/21S ratios, indicating a slight accumulation of late precursors to the 18S rRNA (S1 Fig). In contrast, cells treated with siRNA *HBS1L-*2 showed no significant changes in the expression of any pre-rRNA species relative to controls.

### RNA expression analysis suggestive of involvement of skeletal, urogenital, and eye development pathways

**Fig 5:**
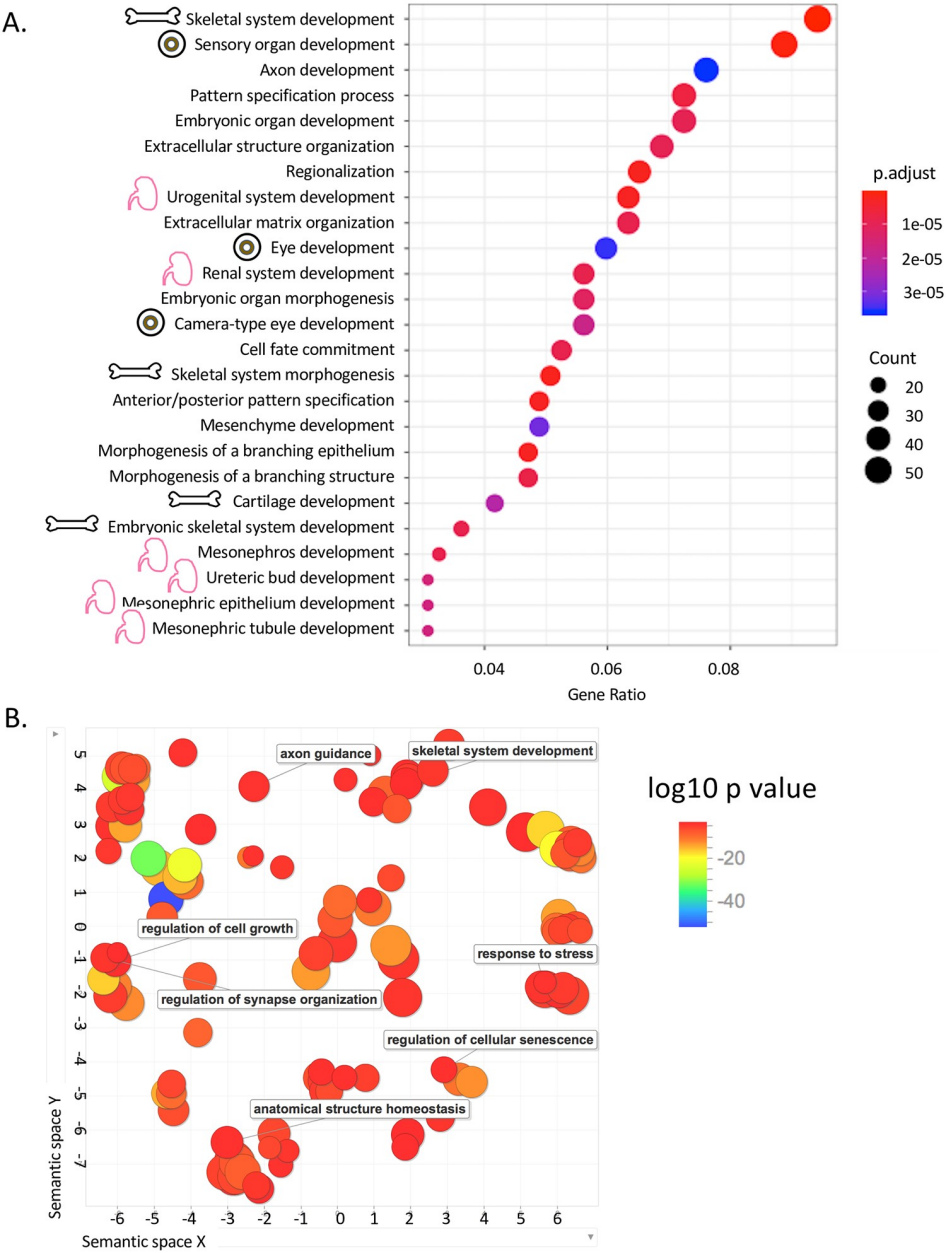
Differential pathway expression by RNASeq. (A) Differences in expression of gene ontology pathways by biological process. Skeletal, eye, and renal pathways were overrepresented and are indicated with cartoons. (B) RNASeq gene ontology map by biological process outputs from REVIGO ontology software.

Patient-derived and control epidermal fibroblasts were used to generate an mRNA library for mRNA-Seq. Principle component analysis (PCA) demonstrated clustering of patient and control samples between technical replicates (S2 Fig). Furthermore, gene coverage was similar between two control fibroblast lines and between independent experimental replicates for both patients and controls. Gene expression analysis for biological processes showed that 1601 of 3730 gene sets were upregulated in patient fibroblasts, with 585 gene sets significant using FDR <25% and 275 gene sets being significantly enriched at p <0.01. 713 gene sets were significant downregulated at FDR <25% and 300 at p<0.01 (S1 Table). Gene ontology assessment demonstrated major changes in biological process pathway expression for many skeletal and renal pathways, as well as sensory pathways, specifically relating to the eye (Fig 5A). In addition, several gene sets relating to organ development were different between patients and controls. Furthermore, the axon development pathway was significantly altered in the patients, which is of interest given the patient’s diffuse weakness without evidence of myopathy.

Cellular component analysis showed that 316 of 488 gene sets were upregulated in patient fibroblasts, with 163 gene sets significant using FDR <25% and 82 gene sets being significantly enriched at p <0.01 (S2 Table). 71 gene sets were significant downregulated at FDR<25% and 35 at p<0.01. By gene ontology assessment, the most differentially expressed groups were positive regulation of biological process, system development, and extracellular matrix organization (Fig 5B, S3 Table).

### Hbs1L-deficient fibroblasts had increased translation efficiency of ribosomal RNA

Patient-derived fibroblasts were grown to near confluence and then treated with cycloheximide to pause translation. mRNA not being actively translated was degraded, and then the ribosomes were removed from the remaining mRNA. The remaining mRNA that had been bound to ribosomes was then sequenced using next generation sequencing. The principle component analysis and expression heatmaps demonstrated excellent reproducibility within biological replicates and marked variance between patient fibroblasts and controls (S3 Fig).

**Fig 6:**
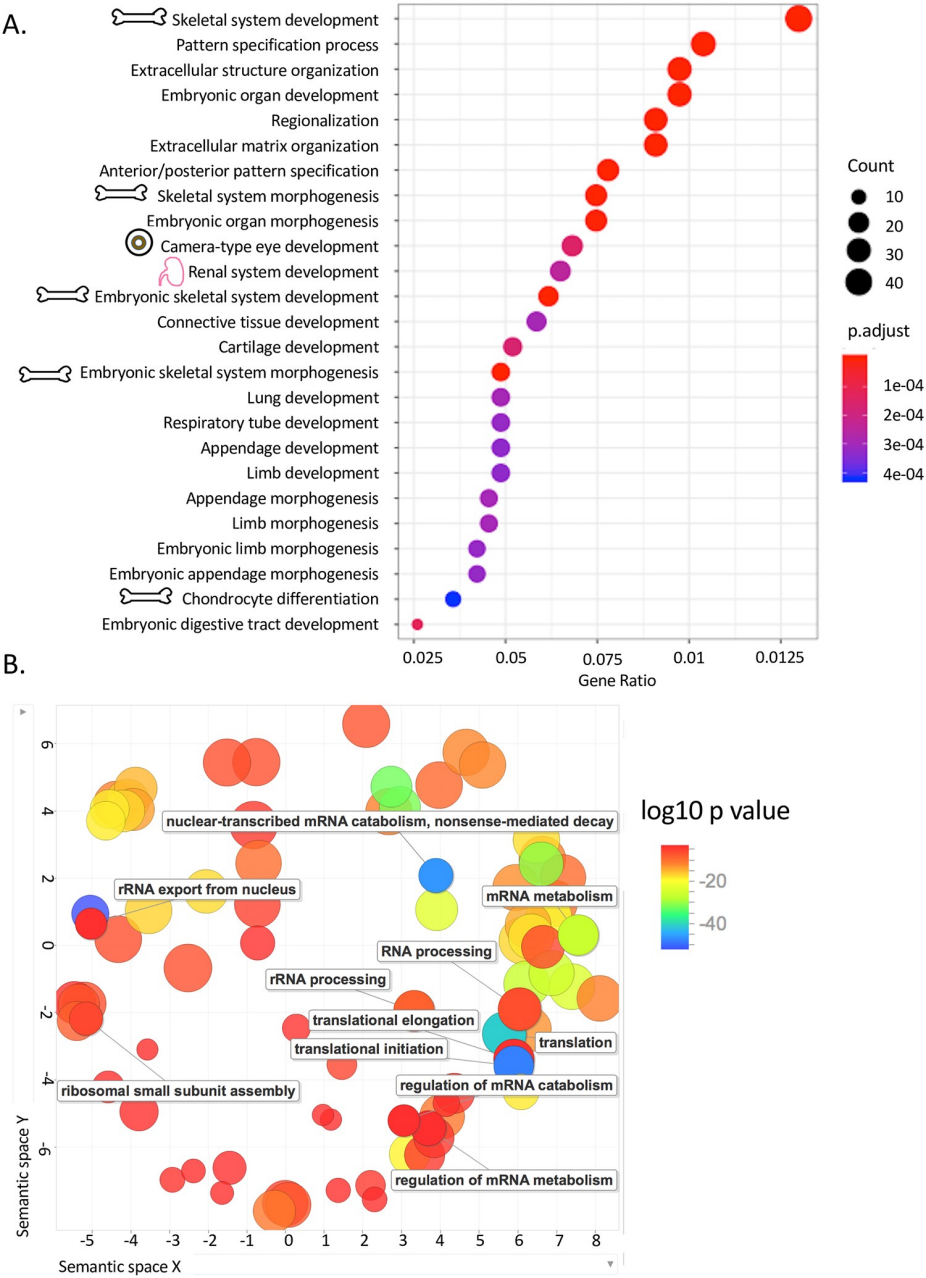
Differential pathway expression by RiboSeq. (A) Differences in expression of gene ontology pathways by biological process. Skeletal pathways were overrepresented while eye and renal less so than the RNASeq results, all are indicated with cartoons. (B) RiboSeq gene ontology map by biological process outputs from REVIGO ontology software.

Ontology analysis of biological processes for the Ribo-Seq data were different than the mRNA-Seq, however skeletal system development and morphogenesis genes remained significantly different in both datasets (Fig 6A). Gene sets relating to embryonic development, extracellular matrix and structures, and limb/appendage development were also different in the patient Ribo-Seq samples compared to controls. Cellular component analysis for the Ribo-Seq data showed the greatest effects in regulation of multicellular processes, anatomical structure morphogenesis, and extracellular matrix organization (Fig 6B).

**Fig 7:**
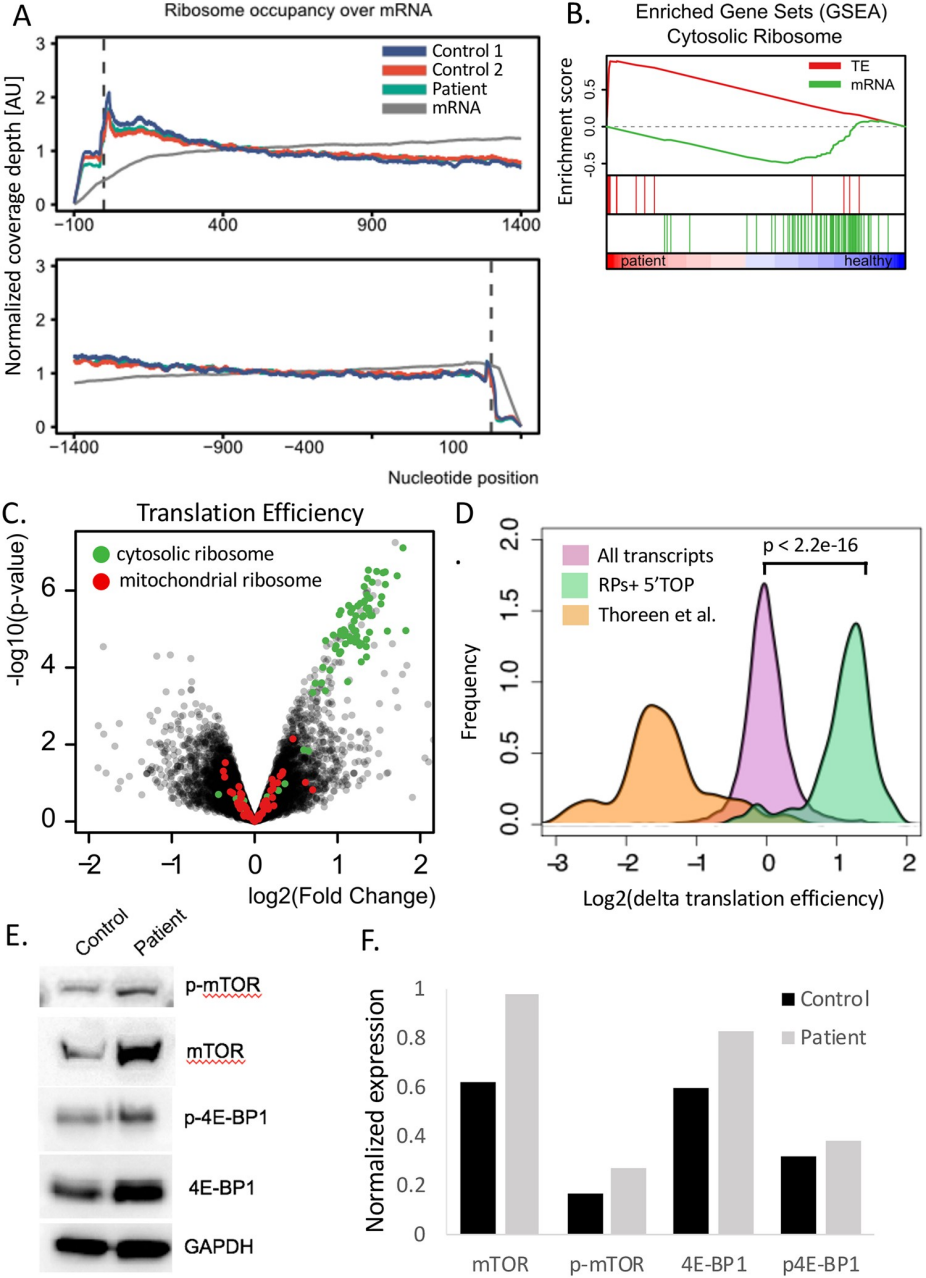
RiboSeq data analysis demonstrates no difference in ribosomal footprint but enhanced translation efficiency of ribosomal RNAs. (A) Ribosome footprinting shows similar location of ribosomes on the mRNA transcripts in patients compared to controls. (B) Gene set enrichment analysis of cytosolic ribosome translation efficiency (red line) and mRNA (gene line) in patients compared to controls. (C) Volcano plot demonstrate translation efficiency (TE) of all genes with overlay of mitochondrial ribosome TE (red) and cytosolic ribosome TE (green). (D) Comparison of translation efficiency in patients looking at all transcripts (purple) compared with known ribosomal protein transcripts and verified 5’TOP transcripts (green). For comparison sake, the TE for the RP + 5’TOP group from the 2012 Thoreen et al. paper using an mTOR inhibitor is also indicated (orange). (E) Western blot showing mTOR, phospho-mTOR, 4-EBP1, and phospho-4EBP1 expression in patient vs control fibroblasts. (F) Quantification of Western blot results in (shown in E.) normalized to GAPDH expression.

Ribosomal footprint analysis, looking at the position of the ribosomes on the mRNA transcripts, showed no differences between patient and control fibroblasts (Fig 7A). We directly assessed the coverage of 15 individual genes and found no differences in coverage individually. Assessment of biological process mapping for the TE showed 1194 of 3323 genes with TE upregulated, 51 gene sets significant at FDR <25% and 50 sets significantly enriched at p<0.01 (S3 Table). Downregulation was significant at FDR <25% for 142 gene sets and at p <0.01 for 109 gene sets. Cellular component analyses showed 236 of 461 gene sets upregulated, with 55 enriched at FDR <25% and another 55 enriched for downregulation with FDR <25% (S4 Table). Gene set enrichment analysis demonstrated marked enhancement of the TE of cytosolic ribosomal RNA in Hbs1L-deficient samples compared with controls (Fig 7B and 7C). The translation of ribosomal RNA and verified 5’TOP genes were upregulated in patient samples compared to controls, in direct contrast to the effects of mTOR inhibitors on this gene set (Fig 7D). Furthermore, assessment of mTOR, phosphorylated mTOR, 4-EBP1, and phosphorylated 4-EBP1 demonstrated increased expression in patient fibroblasts (Fig 7E and 7F). mRNA expression of mTOR in the mRNA-Seq data set was not different between patient and control fibroblasts.

### Hbs1L-deficient mice exhibit a similar phenotype to humans

**Fig 8:**
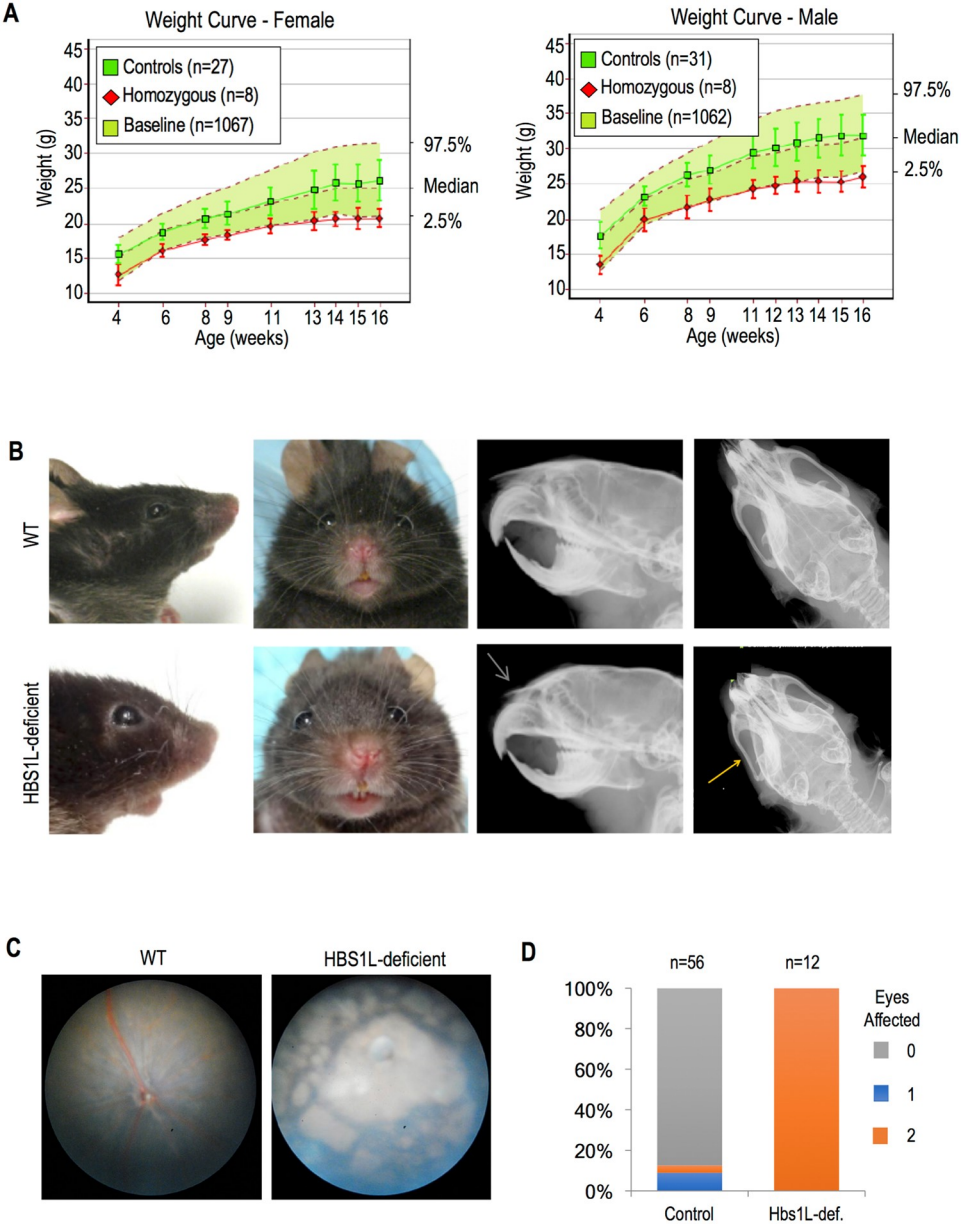
Hbs1L-deficient mice demonstrate growth restriction and facial dysmorphism. (A) Growth curves for female (left panel) and male (right panel) Hbs1L-deficient mice compared with controls and historical baseline. (B) Photographs depicting differences in snout length (first panel) and snout symmetry (second panel) in Hbs1L-deficient versus control mice. Also examples of radiographs comparing anomalies in Hbs1L-deficient mice including shortened nasal bone (green arrow) and zygomatic bone depression (yellow arrow). (C) Representative photos of retinas of wild type and Hbs1L-deficient mice. (D) Distribution plot demonstrating the prevalence of retinal anomalies in Hbs1L-deficient versus control mice.

The *Hbs1l*-KO mice were generated and extensively phenotyped by the Wellcome Sanger Institute (S4 Fig). Both male and female KO mice weighed significantly less than controls at various ages (Fig 8A). The mice also demonstrated abnormal facial structure that was more prevalent in females; 86% of females had depressed nasal bones compared to 43% of males, while another 86% of females had shortened nasal bones compared to only 14% of males (Fig 8B). Other anomalies included malocclusion, depressed zygomatic bones, dental asymmetry, and vertebral defects such as transitional vertebrae and abnormal shape. The ophthalmologic examination revealed atypical retinal pigmentation in the KO mice (Fig 8C and 8D). Additionally, males were found to be uniformly infertile (S5 Table).

### HBS1L-deficient mice display tissue-dependent Pelota depletion

**Fig 9:**
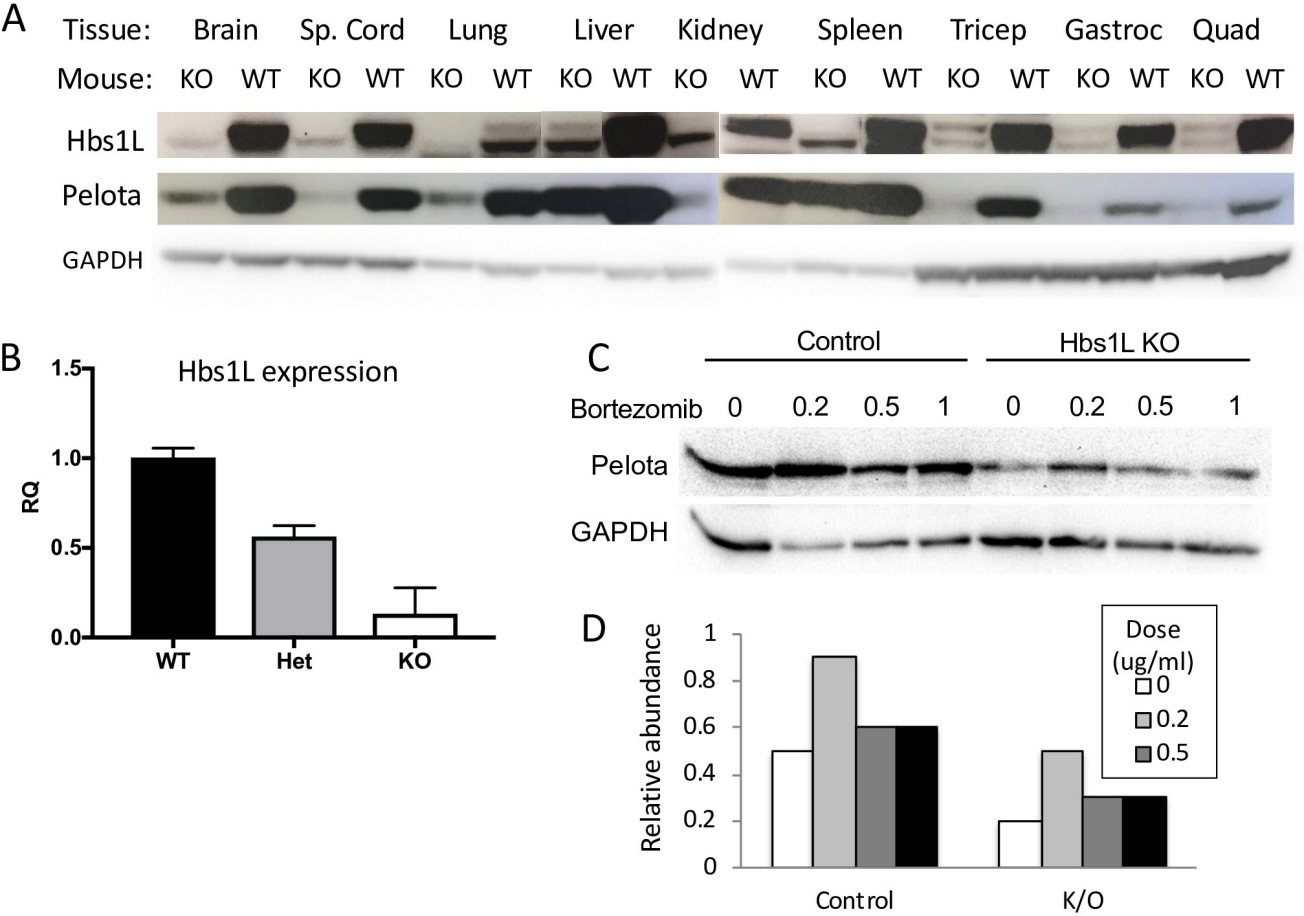
Expression of Hbs1L and Pelota in Hbs1L-deficient mice. (A) Western blot portraying Hbs1L and Pelota protein expression in mouse tissues as indicated. Liver and lung lanes for Hbs1L were spliced due to a loading error, but are otherwise unedited. (B) Relative quantification (RQ) of *HBS1L* transcript expression as determined by RT-PCR in *HBS1L-*KO, heterozygous, and WT mice. Beta-2-microglobulin was used as the housekeeping gene for relative quantification calculations, and mice were analyzed at E9.5. (C) Western blot of Pelota and GAPDH expression in control and Hbs1LKO fibroblasts treated with indicated doses of the proteasome inhibitor bortezomib. (D) Quantification of Pelota band signal in (C) relative to GAPDH expression.

*Hbs1l*-KO mice were assessed for expression of Hbs1L and Pelota in a variety of tissues by Western blot (Fig 9A). Hbs1L levels were drastically reduced in the *Hbs1l*-KO mice relative to controls, though residual expression at the expected band size for Hbs1L-V3 was observed in various tissues. Some residual expression of V1/V2 was also noted on the Western blot, and this is due to an incomplete knock-down with some residual protein expression, making these mice Hbs1L reduced rather than completely null. Indeed, Hbs1L-deficient E9.5 stage embryos displayed 10% residual expression of *Hbs1l* mRNA by RT-PCR (Fig 9B). Pelota expression in the *Hbs1l*-KO tissues was markedly reduced in skeletal muscle, nervous system, and lung tissue compared to WT (Fig 9A). Interestingly, Pelota expression was abundant in the KO liver and spleen, though still reduced from controls. When we treated mouse control and Hbs1L knock-out fibroblasts with a proteasome inhibitor, Pelota protein was increased when the mouse Hbs1L-deficient fibroblasts were treated with the proteasome inhibitor at non-toxic doses (Fig 9C and 9D).

## Discussion

In lower eukaryotes, Hbs1 protein has been established as a critical factor in several translational processes for its ability to bind with Pelota and perform ribosomal recycling. Pelota is a known interacting partner of Hbs1L, and we noted a marked reduction in Pelota protein levels in patient cell lines despite normal *PELO* transcript levels. This effect was also observed in the majority of murine tissues. One explanation for this finding is that Pelota stability is disrupted in the absence of Hbs1L, and destabilization of its tertiary structure leads to premature degradation of the protein *in vivo*. In concordance with this hypothesis, treating both patient fibroblasts and Hbs1L-deficient mouse fibroblasts with a specific dose of proteasome inhibitor induced an increase in Pelota levels. This effect was also observed in control cells, indicating that the proteolytic degradation pathway is responsible for regulating Pelota levels under normal conditions. Similar observations of coordinate regulation at the post-translational level have been described for paralogous translation factors eRF1 and eRF3 [28, 29], affirming a pattern in which trGTPases and their binding partners are targeted for degradation when they are in an unbound state.

Monosomes are traditionally considered inactive complexes, consisting of “vacant couples” of large and small subunits which stably associate in the cytoplasm in the absence of mRNA [30]. When synthetic demand increases, monosomal subunits typically dissociate from one another and then re-associate in the presence of an mRNA transcript to initiate translation. The mechanism by which the subunits are split has not been well-characterized, and, although Pelota:Hbs1L has been shown to engage in ribosomal splitting in other contexts including ribosomal rescue [15, 16, 31] and translational re-initiation after cell stress [17]; our findings suggest that it may also be involved in pre-translational dissociation of cytosolic monosome complexes. Human Hbs1L-deficient fibroblasts displayed increased levels of 80S monosomes relative to controls, suggesting that Hbs1L deficiency may impair translation initiation by preventing the dissociation of monosomes into translationally available subunits. This has been previously shown for yeast Hbs1, where a mutant Dom34 caused increased accumulation of inactive 80S ribosomes both at baseline and following starvation [32]. The heightened baseline monosome peak in patient cells may represent a missing link to the initial rate-limiting step of translation initiation.

Alternatively, the enhanced monosome peak could reflect a population of monosomes that are unable to dissociate due to incomplete salvation from a previous round of aberrant translation or a defect in the ribosome itself. In yeast, aggregated monosomes induced by Dom34 deficiency are resistant to dissociation at low Mg^2+^ concentrations, indicating that they are not affected by the electrophysiological conditions that regulate the natural equilibrium between free and paired ribosomal subunits [33]. We profiled several pre-rRNA species in our patient’s cells to address the possibility of a defective rRNA processing pathway leading to a population of “stuck” monosomes; however, no significant deficits in ribosomal processing were observed. It is likely that Pelota:Hbs1L regulates monosome binding at the post-translational level, either through direct contact with the inactive complex or through critical events that occur during ribosomal rescue from aberrant translation.

Analysis of mRNA-Seq data demonstrated strong correlation with the patient’s clinical phenotype. Skeletal, renal/urogenital, and eye development biological process pathways were all significantly different in patient versus control fibroblasts, corresponding to the clinical phenotype of skeletal, urogenital and ocular anomalies. There was also a significant difference in axon development genes, and this suggests a neuronal etiology of the patient’s diffuse weakness.

Ribo-Seq is a technique that sequences only the RNA being actively transcribed by the ribosome [34–37]. We used Ribo-Seq to assess whether there was clustering of ribosomes at a particular region of the mRNA sequences. For instance, if there is a defect in non-stop decay increased ribosome coverage at the 5’ end of the transcript would be expected. Ribosomal footprint analysis showed no difference in the ribosomal footprint in patient versus control fibroblasts. Hbs1 has been shown in yeast and plant systems to function with Pelota in NSD [8–10]. The footprinting is an indirect assessment of NSD and supports the shRNA data showing no differences in NSD. This difference might be due to differences in the importance of the Hbs1LV1/V2 isoforms for NSD and NGD in the human compared with yeast. However, it is also possible that it is a function of footprinting strategy, as others have recently shown with an epidermal Pelota-deficient model that shorter length sequencing (20–21 nt) revealed differences in footprinting where standard length footprints (28–34 nt) did not [38]. This deserves further investigation.

In addition to the position mapping of the ribosomes, Ribo-Seq was also used to assess translation efficiency by comparing with the mRNA-Seq data. Strikingly, efficiency was dramatically increased for cytosolic ribosomal RNAs compared with control fibroblasts. The ribosomal genes upregulated correspond with the ribosomal RNAs known to be upregulated by mTOR and phosphorylation of 4-EBP [39]. In accordance with this likely mechanism, levels of phosphorylated and native mTOR and 4-EBP proteins were all increased in the Hbs1L deficient fibroblasts compared with controls. This indicates that the enhanced ribosomal translation is likely to be an mTOR mediated response to the diminished ribosomal pool.

*Hbs1l*-KO mice demonstrated abundant Pelota expression in the liver and kidney despite the absence of Hbs1L. In addition, others have shown an important role for Pelota in epidermal regulation [38], and the Hbs1L-deficient patient did not demonstrate any skin phenotype. Tissue-specific binding of Pelota with novel interacting partner GTPBP2 in central nervous tissue has recently been reported [40], and it is possible that another Pelota binding partner exists in the liver, kidney, and skin, making Hbs1L less critical for the health of these tissues. Alternatively, it could be that ribosomal RNA upregulation seen in the Hbs1L-deficient human fibroblasts is not as robust in tissues that demonstrate a phenotype, causing them to be unable to overcome the inability to re-utilize unbound 80S monosomes.

These findings begin to detail an intricate and variegated role for Pelota:Hbs1L in mammalian translational processes. This is further complicated by the isoforms of Hbs1L, and the particular functional differences between Hbs1LV1/V2 and Hbs1LV3. We have demonstrated that Hbs1LV1/V2 deficiency leads to diminished Pelota in mammals. Additionally, we show that deficiency of Hbs1L:Pelota complex leads to increased levels of 80S ribosomal monosomes as well as an increase in translation of ribosomal RNA. This appears to be an mTOR-dependent phenomenon. Fibroblasts from the patient exhibited altered expression of gene families related to skeletal development, renal development, and ocular development, relating well to the clinical phenotype of the patient. Skeletal gene pathways and many developmental pathways were also significantly altered in the Ribo-Seq analysis, indicating differential translation of these gene sets compared to controls. In addition to our biological insights, we have identified proteasome inhibition to be effective in increasing the levels of Pelota in Hbs1L-deficient patients, which may have therapeutic potential.

## Methods

### Ethics statement

The IRB at Boston Children’s Hospital approved the human cell work under the protocol 10-02-0253. The IUCAC at Boston Children’s Hospital approved our mouse work (approval number 16-06-3182R). The work followed the Guide for the Care and Use of Laboratory Animals and all of the regulatory protocols set forth by the Boston Children’s Hospital Animal Resources at Children’s Hospital (ARCH) facility.

### Generation of Hbs1l-deficient mice

Mice with a targeted pre-conditional mutation in *Hbs1l* containing a LacZ reporter allele (“knockout-first” [41]) were obtained from the International Knockout Mouse Consortium (Knockout Mouse Project (KOMP) Repository, IKMC project 79564; http://www.mousephenotype.org/data/alleles/MGI:1891704/tm1a(KOMP)Wtsi), developed by the Wellcome Sanger Institute (Hinxton CB10 1SA, UK) [41–44]. In brief, embryonic stem (ES) cells (line JM8A3.N1 [43]) were cultured according to the standard KOMP protocol and electroporated using the targeting vector HTGRS06009_A_B02 (Fig 7). The targeting vector contained an IRES:LacZ trapping cassette and a floxed promoter-driven Neo-cassette, which was inserted into introns 4–5 of the murine *Hbs1l* gene. FRT sites flanked the LacZ and Neo cassettes and LoxP sites flanked exon 5, which was targeted for its critical ability to create frameshift mutations in *Hbs1l* splice variants V1 and V2 when deleted. Splicing across the gene-trap insertion disrupted *Hbs1l* gene function and rendered a null initial allele (*Hbs1l*^*tm1a*^). ES cells carrying the *Hbs1l*^*tm1a*^ allele (clone EPD0693_2_A02) were injected into C57BL/J blastocysts, which were subsequently implanted into the uterine horns of C57BL/6 mice. Chimeras and subsequent progeny were mated to 6N Taconic wild-type mice for germline production and colony expansion. Heterozygous progeny were cross-bred to produce homozygous knockout-first (*Hbs1l*-KO) mice and wild-type (WT) controls. Mice were quarantined at Charles River Laboratories (Worcester, MA, USA) before import into our animal facility at Boston Children’s Hospital (Boston, MA, USA). Animal study protocols were approved and monitored by the Institutional Animal Care and Use Committee (IACUC) at our institution.

Genotypes were confirmed by PCR using the *Hbs1l* forward primer (Hbs1l_F: 5’-TCTAATTCATGTGTGCCGCC-3’) and reverse primer (Hbs1l_R: 5’-TCCTGTGTTTTACCTGCATAGAGC’3’) which flanked the targeted exon 5, producing the WT PCR product of 483 bp. Addition of the targeting cassette specific primer (Hbs1l_Cas: 5’-TCGTGGTATCGTTATGCGCC-3’) to the PCR master mixes to induce multiplex reactions generated a mutant-specific allele of 338 bp.

### Phenotyping of Hbs1L-deficient mice

Mouse phenotyping procedures were performed by the Wellcome Sanger Institute (WSI) in accordance with the protocols described under the MGP Select Pipeline (http://www.mousephenotype.org/impress/procedures/15). Body weight was measured twice weekly in *Hbs1l*-KO and WT mice between three and sixteen weeks of age. Further phenotyping measures included hematology, eye morphology, fertility, morphology, clinical chemistry, and body composition.

### Culturing of human cell lines

Patient-derived human dermal fibroblasts (cell line 128–01) were harvested from skin biopsy samples using standard methods. Three fibroblast control cell lines (59 or “control 1”, 2104 or “control 2”, and 2127 or “control 3”) were obtained from our stored cell lines and our collaborators. Fibroblasts were grown to roughly 80% confluency in DMEM media with 15% FBS and penicillin-streptomycin glutamate in vertical T75 flasks. Cells were lifted by trypsin digestion and pelleted at 1000 rpm at room temperature for 8 minutes (Allegra X-12R centrifuge with SX4750A rotor, Beckman Coulter, Brea, CA, USA).

EBV-transformed cells (lymphoblastoid cell lines, or LCLs) were developed by co-culture of competent EBV with patient-derived monocytes as previously described [45]. Two human LCL control cell lines (Ncc and N2) were obtained from our collaborators. LCLs were grown in RPMI 1640 media with 15% FBS and penicillin-streptomycin glutamate in vertical T75 flasks. Cells were grown in suspension and pelleted by centrifugation at 1300 rpm for 10 minutes at room temperature (Allegra X-12R centrifuge with SX4750A rotor Beckman Coulter).

### RT-PCR analyses

For human mRNA extraction, patient and control fibroblasts were trypsinized and pelleted (Allegra X-12R centrifuge with SX4750A rotor, Beckman Coulter) at 1000 RPM for eight minutes at room temperature, and mRNA was isolated from cell pellets using the AllPrep DNA/RNA Mini Kit (Qiagen, Valencia, CA, USA). For mouse extraction, gastrocnemius muscles were removed from euthanized animals, frozen in cold methylbutane and stored at -80C for several months prior experimentation. mRNA was extracted from frozen skeletal muscle tissue using the RNeasy fibrous tissue mini kit (Qiagen) according to the manufacturer’s instructions. For both human and mouse samples, mRNA was reverse transcribed into cDNA using the SuperScript III First-Strand Synthesis System (Invitrogen, Carlsbad, CA, USA) and genomic DNA was eliminated using the RNase-Free DNase set (Qiagen).

Experimental replicates for RT-PCR contained 80 ng of human or mouse cDNA combined with appropriate primers (described in S6 Table) and Fast SYBR Green (Invitrogen) according to the manufacturer’s instructions. Reactions were run under standard conditions using the ABI Step One Plus qPCR machine (Thermo Fisher Scientific, Waltham, MA, USA) and analyzed using the StepOne Real-Time PCR System (Thermo Fisher Scientific).

Tissue from E9.5 embryos was homogenized and total RNA was extracted as above. 200 ng of extracted RNA was used in a 10 μl reaction using the TaqMan RNA-to-CT One-Step Kit (Applied Biosystems, Foster City, CA, USA). TaqMan probe Mm03807425_g1 (Applied Biosystems), flanking the Hbs1l splice acceptor region between exons 4 and 5 (Ensembl Transcript ID: ENSMUST00000219915.1), was used with primer-limited B2m endogenous control (Applied Biosystems) in a multiplex reaction. Reactions were performed in triplicate using the Viia 7 Real-Time PCR System (Applied Biosystems).

### Western blot analyses

Human protein isolates were prepared from fibroblasts and LCLs in lysis buffer, and mouse protein isolates were prepared from homogenized muscle and organ tissues that were boiled at 100°C for five minutes. Protein samples were electrophoresed through 4–12% NuPage Bis-Tris Gel (Thermo Fisher Scientific) at 200V for 50–60 minutes and then transferred onto a PVDF membrane overnight at 10V and 4°C. Membranes were blocked in blocking solution (5% blocking-grade non-fat dry milk in 1X TBS Tween-20 (TBS-T)) for one hour and then incubated overnight at 4°C with primary antibody diluted in blocking solution at the following concentrations: rabbit anti-Hbs1L (10359–1 AP, Proteintech Group Inc., Chicago, IL, USA), 1:500 for human and mouse; rabbit anti-Pelota (ab140615, Abcam, Cambridge, UK), 1:2,000 for human; rabbit anti-Pelota (10582-1-AP, Proteintech Group Inc.), 1:1,000 for mice; and rabbit anti-ABCE1 (ab32270, Abcam), 1:1,000 for human. The following antibodies were used at 1:500 concentration for 2 hour incubation: Phospho-4E-BP1 (Thr37/46) 236B4 (#2855 Cell Signaling,) 4E-BP1 (53H11) (#9644, Cell Signaling); Phospho-mTOR (Ser2481) (#2974, Cell Signaling); mTOR (7C10) (#2983, Cell Signaling). Membranes were then washed three times for ten minutes in TBS-T and incubated with HRP-conjugated goat anti-rabbit secondary antibody (ab6721, Abcam) at a concentration of 1:2,000 in blocking buffer at room temperature for one hour, then subjected to three more ten-minute washes in TBS-T and developed using SuperSignal West Pico Chemiluminescent Substrate (Fisher Scientific, Hampton, NH, USA) for ten minutes. Developed membranes were imaged on an Image Station 440 (Kodak DS; Eastman Kodak Co., Rochester, NY, USA). After imaging, membranes were stripped and incubated with mouse anti-GAPDH primary antibody (ab8245, Abcam) diluted in blocking buffer to a concentration of 1:10,000 for human (1:5000 for the phosphorylation mTOR and 4-EBP assays) and 1:4,000 for mice for one hour at room temperature, and then developed and imaged following the same procedure as previously described. Protein expression data was analyzed using Quantity One software (v. 4.2.1; Bio-Rad Laboratories, Hercules, CA, USA).

### Proteasome inhibition assay

Patient and control fibroblasts, and separately Hbs1L knock out and control mouse fibroblasts, were treated with the 20S proteasome inhibitor Bortezomib at a dose of either 0 mg/mL (conditional controls), 0.2 mg/mL, 0.5 mg/mL, or 1.0 mg/mL. Protein isolates were prepared from treated cells 40 hours after Bortezomib administration and Pelota protein expression was analyzed by Western blot using the same methods described previously.

### Northern blot analyses

Patient and control LCLs were pelleted at 1300 RPM (Allegra X-12R centrifuge with SX4750A rotor, Beckman Coulter) for 10 minutes at room temperature. Total RNAs were extracted with TRIzol reagent (Invitrogen) from cell pellets containing 10–20 x 10^6^ cells. The aqueous phase was further extracted with phenol-chloroform-isoamyl alcohol (25:24:1; Sigma), then with chloroform. Total RNAs were recovered after precipitation with 2-propanol.

For Northern blot analyses, RNAs were dissolved in formamide, denatured for 10 minutes at 70°C and separated on a 1.1% agarose gel containing 1.2% formaldehyde and 1X Tri/Tri buffer (30 mM triethanolamine, 30 mM tricine, pH 7.9) (3 μg of RNAs per lane). RNAs were transferred to a Hybond N^+^ nylon membrane (GE Healthcare, Orsay, France) by passive transfer. After UV cross-linking, pre-hybridization of the membrane was performed for 1 hour at 45°C in 6X SSC, 5X Denhardt’s solution, 0.5% SDS, 0.9 g/ml tRNA. The 5’-radiolabeled oligonucleotide probe was incubated overnight at 4°C. The sequences of the RNA probes were: 5’-ITS1 (5’-CCTCGCCCTCCGGGCTCCGTTAATGATC-3’), ITS2 (ITS2b: 5'-CTGCGAGGGAACCCCCAGCCGCGCA-3' and ITS2d/e: 5'-GCGCGACGGCGGACGACACCGCGGCGTC-3'), 18S (5'-TTTACTTCCTCTAGATAGTCAAGTTCGACC-3'), 28S (5'-CCCGTTCCCTTGGCTGTGGTTTCGCTAGATA-3'). Membranes were subjected to two ten-minute washes in 2X SSC with 0.1% SDS and one ten-minute wash in 1X SSC with 0.1% SDS and then exposed. Signals were acquired with a Typhoon Trio PhosphorImager (GE Healthcare, Boston, MA, USA) and quantified using the MultiGauge software (Fujifilm, Tokyo, Japan).

The assay was repeated using siRNA-treated HeLa cells. Two 19-mer siRNAs (Eurogentec, Seraing, Belgium) were used to silence expression of the HBS1L mRNAs in HeLa cells. The first siRNA (HBS1L-1: 5’-GCAGUUCUGAAGAACAAGUdTdT-3’) targeted all three HBS1L splice variants, whereas the second (HBS1L-2: 5’-CCAGUAGAUUCCCAGACAUdTdT-3’) targeted V1 and V2 mRNAs only. The efficiency of both probes was verified by Western blot analysis. Each siRNA solution was added at a final concentration of 500 nM to 200 μl of cell suspension (50 x 10^6^ cells/ml diluted in Na phosphate buffer, pH 7.25, containing 250 mM sucrose and 1 mM MgCl_2_). Electro-transformation was performed at 240 V with a Gene Pulser (Bio-Rad, Hercules, CA, USA). Control HeLa cells were electro-transformed with a scramble siRNA (siRNA-negative control duplex; Eurogentec, Liège, Belgium). After a ten-minute incubation at room temperature, cells were plated and grown in in DMEM Gibco media (Thermo Fisher Scientific) supplemented with 10% fetal bovine serum and 1 mM sodium pyruvate (Sigma-Aldrich, St. Louis, MO, USA) at 37°C for 48 hours. Total RNA was then isolated, analyzed by Northern blot, and quantified using the same procedures as previously described.

### Polysome profiling

For glucose starvation assays, media was removed from patient and control fibroblast cultures at roughly 75% confluence and replaced with either DMEM with 15% FBS (fibroblast standard growth media) or DMEM without FBS (fibroblast starvation media). This assay was repeated in patient and control LCLs using RPMI with 15% FBS (LCL standard growth media) and RPMI without FBS (LCL starvation media). Antibiotics were not added at this stage to diminish any potential interference with ribosomal activity. Cells were then grown to near-confluence (roughly 80–90%), as completely confluent cells will down-regulate translation. Cell lysates were prepared from fibroblasts after 24 hours in starved or fed conditions and polysome profiles were assessed by previously published methods [2, 3, 15, 46].

Cells were washed once with the ice-cold buffer: 20 mM Tris-HCl, 50 mM NaCl, 50 mM KCl, 10 mM MgCl_2_, 0.1 mg/ml cycloheximide while attached to the cell culture plate. Lysis was done in the same buffer supplemented with 1% Triton X100, protease inhibitors (Roche), 1 mM DTT, and 40 U/ml Superase-In (Thermo Fisher Scientific). Cell debris was removed by the centrifugation at 12.000 g for 4 min. Cleared lysates were loaded on 10–50 sucrose gradients and spun 3 hours at 35.000 g. For ribosome profiling, the concentration of magnesium was lowered to 5 mM, lysates were pre-treated with 1000 U RNase T1 (Epicentre) for 30 min at room temperature, and the monosomal peak was collected for further Ribo-seq assay. There was no sign of ribosomal degradation and over digestion as a result of such treatment.

### mRNA-Seq library preparation and sequencing

Total RNA was isolated from aliquots of the same lysates used for ribosomal profiling. Trizol LS (Thermo Fisher Scientific) followed by the Direct-zol kit (Zymo Research) ware used for RNA extraction. The library was prepared by the Dana Farber Next Generation Sequencing Core Facility using a Kapa library prep kit. Sequencing was done on an Illumina NextSeq 500 using single-end 75bp reads.

### Ribo-seq library preparation and sequencing

Ribosome protected footprints were isolated from sucrose gradient fractions with Trizol LS (Thermo Fisher Scientific) and Direct-Zol kit (Zymo Research). Libraries were prepared as described previously [47]. Briefly, ribosomal footprints (25–32 nt) were cut and eluted from the polyacrylamide gel, the preadenylated adapter was ligated to footprints and reverse transcription performed with individually barcoded primers. After PCR amplification, libraries were pooled together and sequenced on Illumina high-throughput sequencer.

### Sequencing data analysis

An analytical pipeline including calling differentially expressed genes, gene set enrichment analysis, estimating translation efficiencies, et cetera is available here: https://github.com/germaximus/OConnell_2018. Raw sequencing files are accessible from NCBI Gene Expression Omnibus database: GSE123564

### Gene ontology analyses

For ontology analyses, ranked gene sets were uploaded to GOrilla gene ontology assignment software (http://cbl-gorilla.cs.technion.ac.il) and output parameter was selected as process or component depending on the gene list uploaded (biological process versus cellular component) [48, 49]. Outputs from GOrilla were then uploaded to REViGO (http://revigo.irb.hr) and the default medium allowed similarity was used [50].

### Statistical approach

Data are reported as mean ± standard error of the mean (SEM) unless indicated otherwise. Protein expression data derived from Western blots were normalized to the loading control and then calculated as fold change relative to the expression level of the experimental control(s). Relative quantification of gene expression data derived from RT-PCR experiments was performed using the 2^-ΔΔCT^ method. rRNA and pre-rRNA expression data derived from Northern blots were calculated as log_2_ ratios of product to precursor pre-rRNA species and 18S to 28S rRNAs relative to three control cell lines. The single-source nature of our biological sample representing human Hbs1L-deficiency precluded the use of tests of statistical significance in some of our human experimental analyses, though several control samples and a minimum of three technical replicates for each sample were used for all human cell line experiments to increase the power. When replicates were sufficient, as for polysome profiling of fibroblast cultures, we used paired t test to compare patient to control replicates. Statistical analyses for RNA Seq and RiboSeq analyses were calculated using the Github GSEA package in R. Gene ontology significance in GOrilla was assessed using the minimum-hypergeometric (mHG) statistic in R and p values were assigned to be >10^^-3^. For mouse phenotyping, morphological and X-ray data were analyzed using the Fisher Exact Test framework, and body compositional, hematological, and clinical chemistry data were analyzed using the Mixed Model framework (linear mixed-effects model, equation with or without weight). Fertility results were supplied as data.

## Supporting information

S1 Fig Ribosomal RNA and polysome profiles are similar in siRNA Hbs1L-knockdown and control HeLa cells. (A) Northern blot analysis of total RNAs extracted from HeLa cells treated with siRNA *HBS1L-*1 (*HBS1L-*V1, V2, and V3 knockdown), *HBS1L*-V2 (*HBS1L-*V1 and V2 knockdown only), or controls (scramble siRNA). Pre-rRNA species were evidenced with 5’ITS1 and ITS2 probes, revealing precursors to the 18S rRNA (component of the small ribosomal subunit) and the 5.8S and 28S rRNAs (components of the large ribosomal subunit), respectively. (B) Pre-rRNAs were quantified and log_2_ ratios of product to precursor pre-rRNA species, or 18S to 28S rRNAs, were calculated for cells treated with *HBS1L*-1 and *HBS1L-*2 siRNAs. The data were obtained from 3 independent experiments (± S.E.M.). (C) Western blot analysis of total protein extracts from cells treated with scramble, *HBS1L*-1, or *HBS1L-*2 siRNAs shown in A. Hbs1L levels were assessed relative to an α-actin loading control.
(TIFF)

S2 Fig Quality control analysis of RNASeq. A.) PCA showing clustering of patient replicates (red, 5 replicates from 2 separate experiments) and controls (green and blue, 2 replicates per control). B.) Alignment heat map demonstrating similarity of transcripts between patients and controls.
(TIFF)

S3 Fig Quality control analysis of RiboSeq. A.) PCA showing clustering of patient replicates (red and green, 3 replicates each from 2 separate experiments) and controls (purple and blue, 3 replicates per control). B.) Alignment heat map demonstrating similarity of transcripts between patients and controls.
(TIFF)

S4 Fig Targeting vector design for Hbs1l-KO mouse model. Targeting vector HTGRS06009_A_B02 was used for the electroporation of ES stem cells in the generation of *Hbs1l*-KO mice. The vector contained an IRES:LacZ trapping cassette and a floxed promoter-driven Neo-cassette, which was inserted into introns 4–5 of the murine *Hbs1l* gene. FRT sites flanked the LacZ and Neo cassettes and LoxP sites flanked critical exon 5. Splicing across the gene-trap insertion yielded a null allele. Image retrieved from: https://www.komp.org/ProductSheet.php?cloneID=701187.
(TIFF)

S1 Table Gene set enrichment analysis for mRNA expression by RNASeq for biological process. (CSV)

S2 Table Gene set enrichment analysis for mRNA expression by RNASeq for cellular component. (CSV)

S3 Table Gene set enrichment analysis for mRNA expression by translation efficiency for biological process. (CSV)

S4 Table Gene set enrichment analysis for mRNA expression by translation efficiency for cellular component. (CSV)

S5 Table Mating chart demonstrating infertility of male *Hbs1l*-KO mice. (TIFF)

S6 Table Primers used for human and mouse RT-PCR analyses. (TIFF)
