# Supplementary figures and images for: Mammalian Hbs1L deficiency causes congenital anomalies and developmental delay associated with Pelota depletion and 80S monosome accumulation
Source: PLoS Genet. 2019 Feb 1;15(2):e1007917. doi: 10.1371/journal.pgen.1007917 (PMC6373978; doi:10.1371/journal.pgen.1007917)

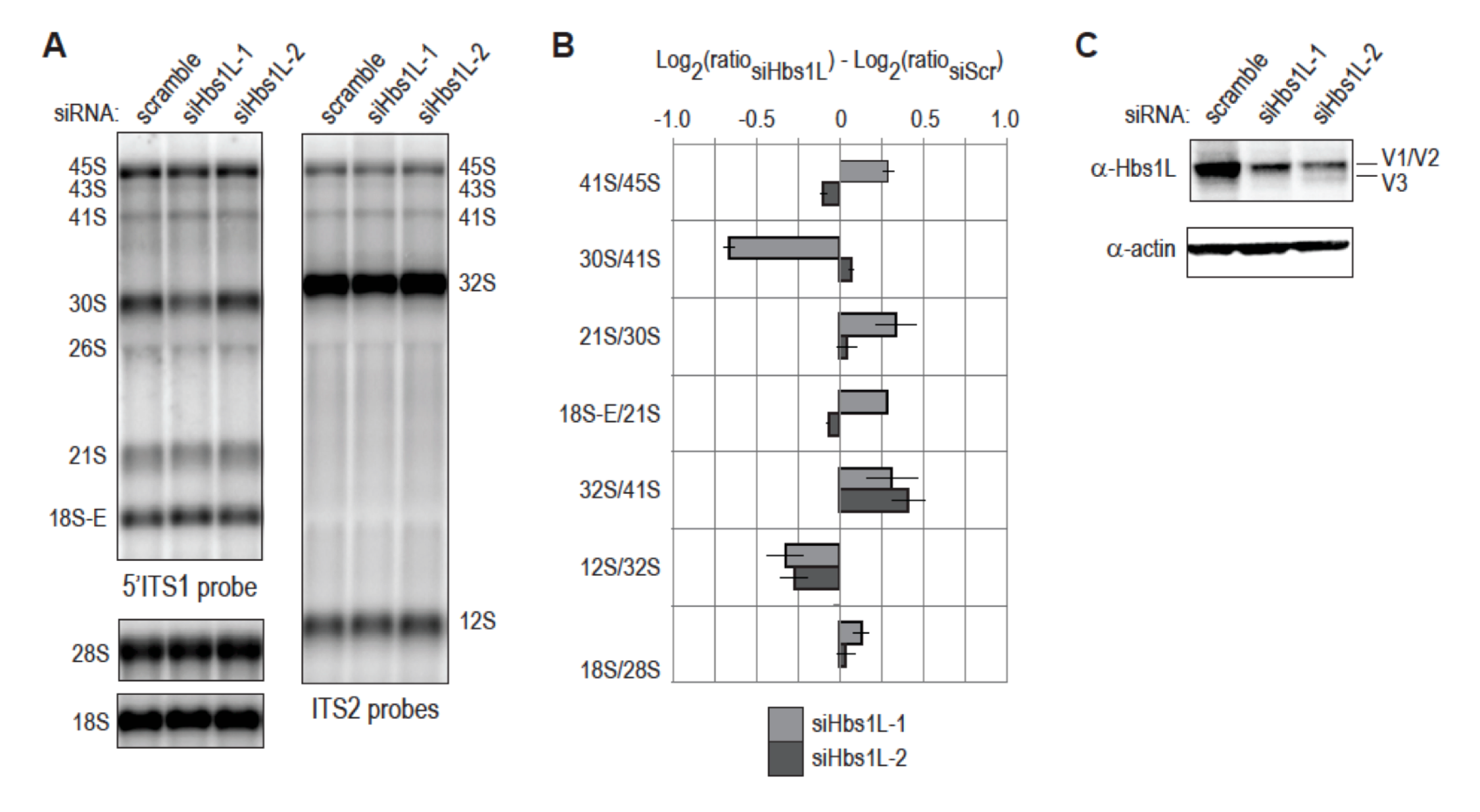

Supplement: S1 Fig — (A) Northern blot analysis of total RNAs extracted from HeLa cells treated with siRNA HBS1L-1 (HBS1L-V1, V2, and V3 knockdown), HBS1L-V2 (HBS1L-V1 and V2 knockdown only), or controls (scramble siRNA). Pre-rRNA species were evidenced with 5’ITS1 and ITS2 probes, revealing precursors to the 18S rRNA (component of the small ribosomal subunit) and the 5.8S and 28S rRNAs (components of the large ribosomal subunit), respectively. (B) Pre-rRNAs were quantified and log2 ratios of product to precursor pre-rRNA species, or 18S to 28S rRNAs, were calculated for cells treated with HBS1L-1 and HBS1L-2 siRNAs. The data were obtained from 3 independent experiments (± S.E.M.). (C) Western blot analysis of total protein extracts from cells treated with scramble, HBS1L-1, or HBS1L-2 siRNAs shown in A. Hbs1L levels were assessed relative to an α-actin loading control. (TIFF) [file pgen.1007917.s001.tiff]

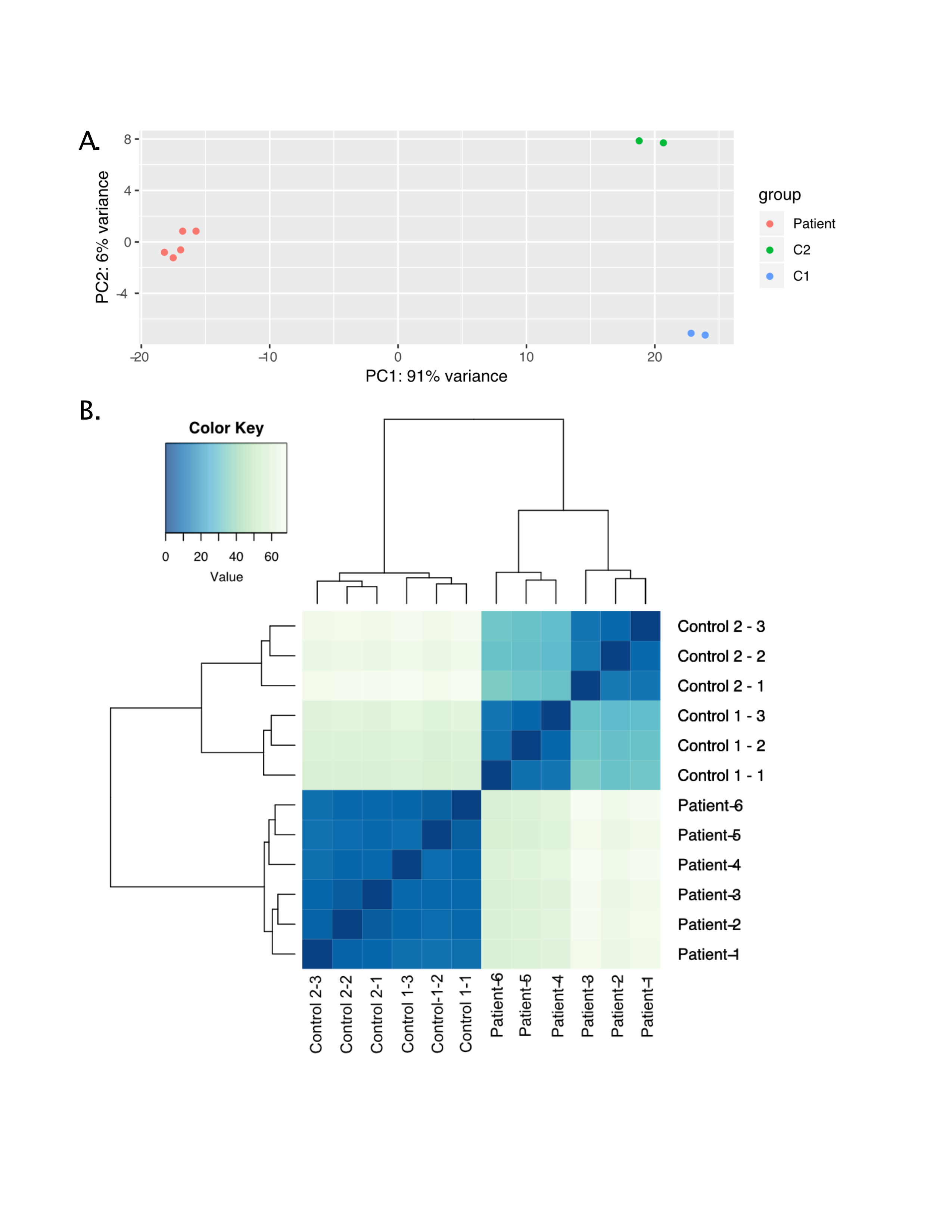

Supplement: S2 Fig — A.) PCA showing clustering of patient replicates (red, 5 replicates from 2 separate experiments) and controls (green and blue, 2 replicates per control). B.) Alignment heat map demonstrating similarity of transcripts between patients and controls. (TIFF) [file pgen.1007917.s002.tiff]

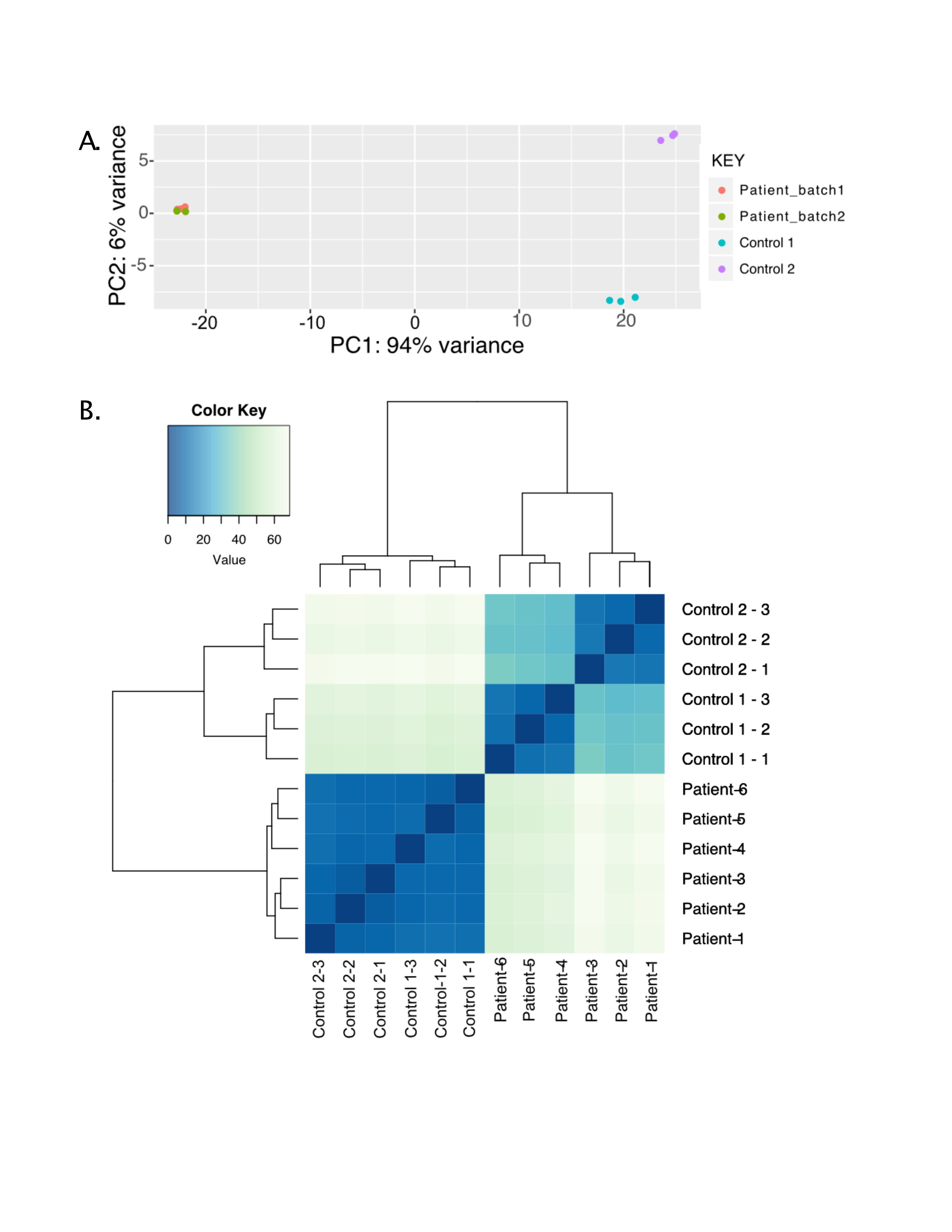

Supplement: S3 Fig — A.) PCA showing clustering of patient replicates (red and green, 3 replicates each from 2 separate experiments) and controls (purple and blue, 3 replicates per control). B.) Alignment heat map demonstrating similarity of transcripts between patients and controls. (TIFF) [file pgen.1007917.s003.tiff]

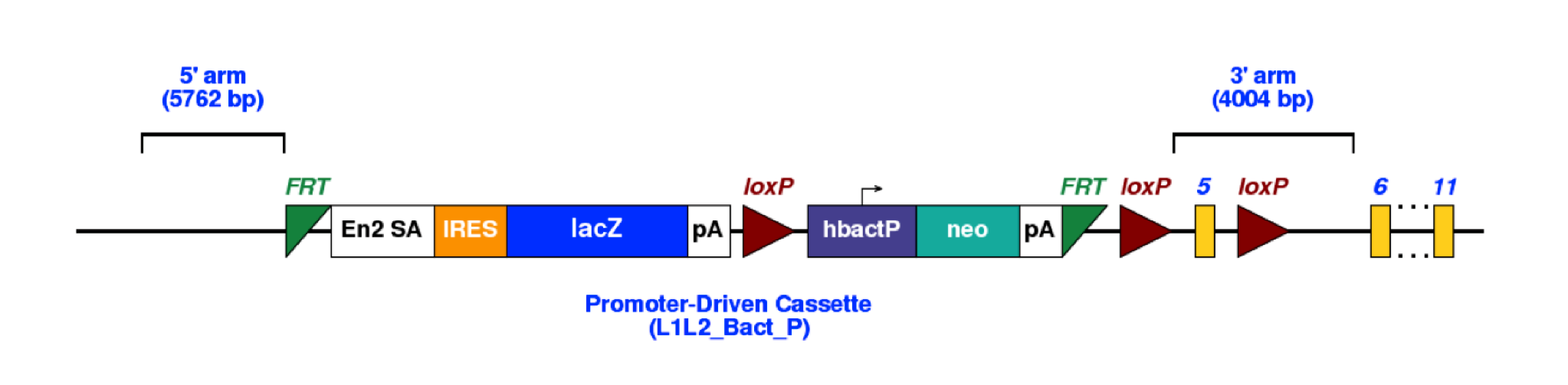

Supplement: S4 Fig — Targeting vector HTGRS06009_A_B02 was used for the electroporation of ES stem cells in the generation of Hbs1l-KO mice. The vector contained an IRES:LacZ trapping cassette and a floxed promoter-driven Neo-cassette, which was inserted into introns 4–5 of the murine Hbs1l gene. FRT sites flanked the LacZ and Neo cassettes and LoxP sites flanked critical exon 5. Splicing across the gene-trap insertion yielded a null allele. Image retrieved from: https://www.komp.org/ProductSheet.php?cloneID=701187. (TIFF) [file pgen.1007917.s004.tiff]

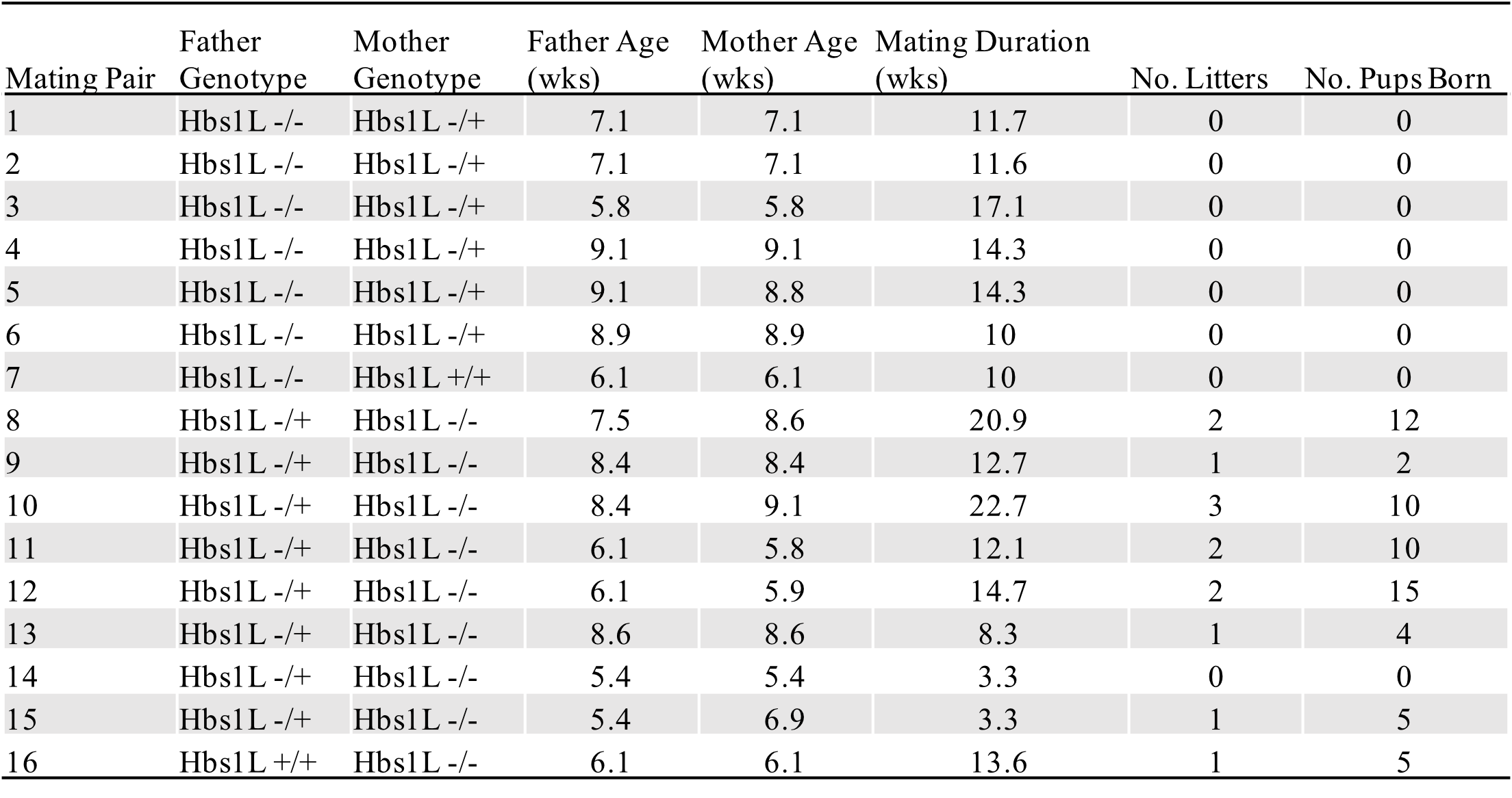

Supplement: S5 Table — (TIFF) [file pgen.1007917.s009.tiff]

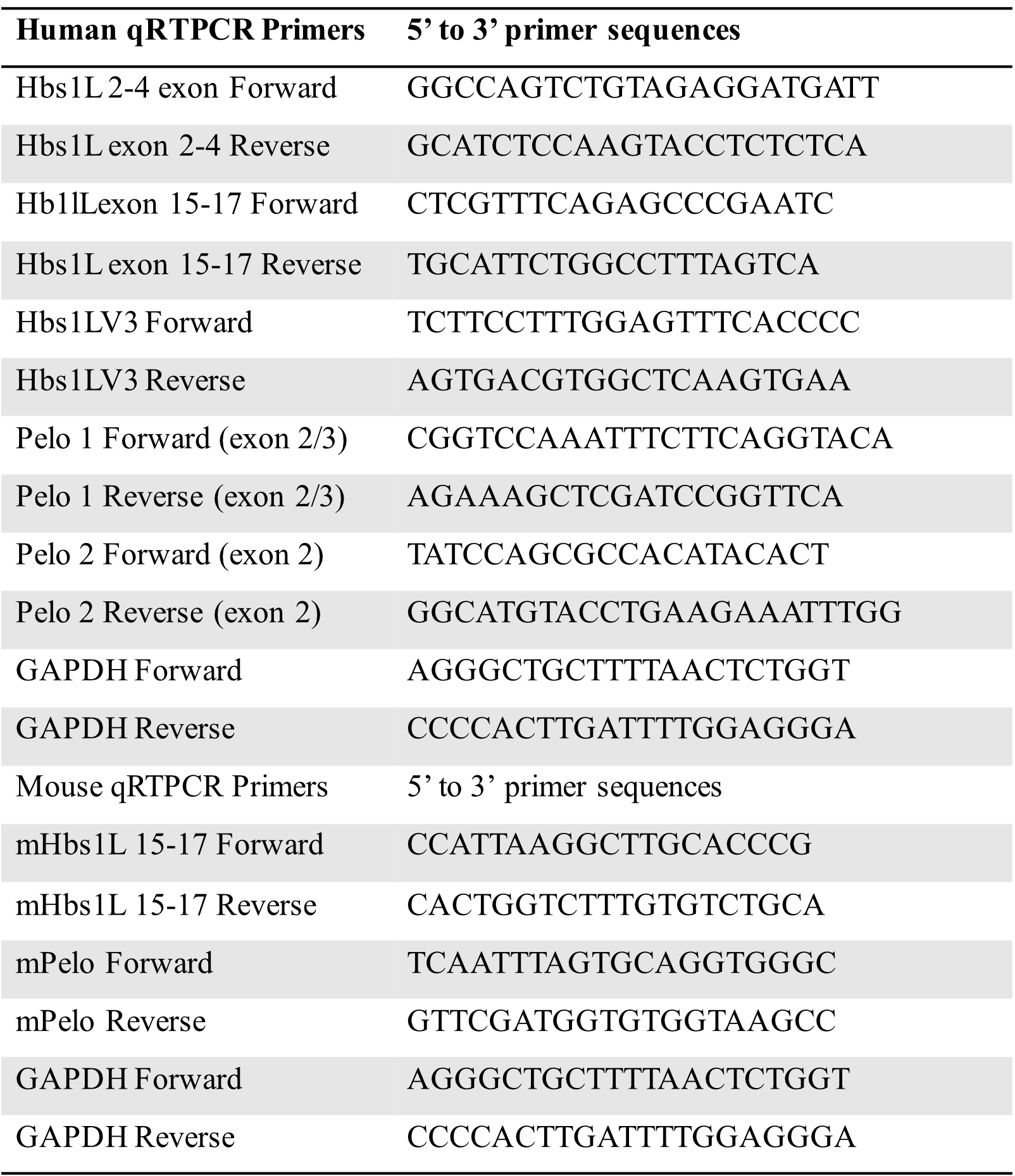

Supplement: S6 Table — (TIFF) [file pgen.1007917.s010.tiff]
